# Zebrafish Larvae Position Tracker (Z-LaP Tracker): a high-throughput deep-learning behavioral approach for the identification of calcineurin pathway-modulating drugs using zebrafish larvae

**DOI:** 10.1038/s41598-023-30303-w

**Published:** 2023-02-23

**Authors:** Sayali V. Gore, Rohit Kakodkar, Thaís Del Rosario Hernández, Sara Tucker Edmister, Robbert Creton

**Affiliations:** 1 Department of Molecular Biology, Cell Biology and Biochemistry, Brown University, 185 Meeting Street, Providence, RI 02912 USA; 2 Center for Computation and Visualization, Brown University, Providence, RI USA

**Keywords:** Drug discovery, Neuroscience

## Abstract

Brain function studies greatly depend on quantification and analysis of behavior. While behavior can be imaged efficiently, the quantification of specific aspects of behavior is labor-intensive and may introduce individual biases. Recent advances in deep learning and artificial intelligence-based tools have made it possible to precisely track individual features of freely moving animals in diverse environments without any markers. In the current study, we developed Zebrafish Larvae Position Tracker (Z-LaP Tracker), a modification of the markerless position estimation software DeepLabCut, to quantify zebrafish larval behavior in a high-throughput 384-well setting. We utilized the high-contrast features of our model animal, zebrafish larvae, including the eyes and the yolk for our behavioral analysis. Using this experimental setup, we quantified relevant behaviors with similar accuracy to the analysis performed by humans. The changes in behavior were organized in behavioral profiles, which were examined by K-means and hierarchical cluster analysis. Calcineurin inhibitors exhibited a distinct behavioral profile characterized by increased activity, acoustic hyperexcitability, reduced visually guided behaviors, and reduced habituation to acoustic stimuli. The developed methodologies were used to identify ‘CsA-type’ drugs that might be promising candidates for the prevention and treatment of neurological disorders.

## Introduction

Zebrafish larvae are rapidly emerging as an excellent model for performing high-throughput drug screening to identify drugs important in normal and/or pathological behaviors^1^ and model complex brain disorders^2–4^. Zebrafish larvae are small and can be studied in large numbers, making them a suitable model for conducting high-throughput screens. Furthermore, a fully characterized genome, availability of genetic mutant lines, and ease of molecular manipulations make zebrafish well suited for studying mechanisms underlying neurological disorders^5^. Zebrafish, from very early developmental stages, show a robust range of complex behaviors—from foraging to learning^6^, similar to those behaviors observed in various mammalian species, suggesting evolutionary conserved mechanisms underlying these behaviors^7^. While various molecular and genetic tools are well established in zebrafish larvae^8–10^, the computational tools for quantifying complex behaviors are still limited and less applicable to high-throughput behavioral screens^11,12^.

Studying animal behavior is a crucial element to understand neural function^13,14^. In particular, accurate quantification of behavior is an important factor for understanding complex behaviors. Recent advances in high-quality video cameras and the presence of commercially available imaging equipment to track an animal’s path of movement and measure specific behaviors have greatly improved our understanding of animal behaviors^15^. Most of the commercially available video tracking solutions are expensive, less flexible in incorporating quantification of multiple behaviors, and may introduce more variability due to manual post-imaging analysis^16^. Most of the currently available methods of behavioral analysis rely on the tracking of animals using a marker or background subtraction image tracing^16^. The requirement of markers for tracking animals is often invasive, cumbersome, and only applicable to certain animal models while background subtraction image tracking methods are sensitive to diverse background settings^17^. There is a need to develop machine learning-based deep-learning methods to define and quantify subtle behaviors that might be missed by the human eye^18^. Present-day advances in computer vision have greatly influenced how animal behavior can be quantified and opened new avenues for developing deep-learning approaches to study behavior^17^.

In the current study, using DeepLabCut modification, we developed a novel deep-learning approach—Z-LaP Tracker, to study a range of behaviors in a high-throughput 384-well format. The developed methodologies were used for the analysis of two prior datasets with images of zebrafish larval behavior after exposure to the calcineurin inhibitors cyclosporine and tacrolimus^19^ and after exposure to 190 FDA-approved drugs^20^.

Calcineurin is a serine/threonine phosphatase known to act in various organ systems, including the nervous system. In the brain, it is involved in modulating synaptic plasticity, learning, and memory^21–23^. Increased calcineurin signaling in the brain is associated with the onset of neurological disorders and calcineurin inhibitors are considered potential therapeutics for treating these disorders^24–28^. Particularly in Alzheimer’s disease, it is hypothesized that a prolonged increase in calcium levels leads to the activation of calcineurin which in turn activates various signaling pathways downstream, resulting in the onset of Alzheimer’s disease^29–31^. This is the reason why calcineurin inhibitors are considered potential drugs for treating Alzheimer’s disease. Supporting the role of the calcineurin signaling pathway in Alzheimer’s disease is a human population study of transplant patients who were treated chronically with calcineurin inhibitors (like cyclosporin and tacrolimus) showed a significantly reduced incidence of AD/dementia in comparison to the general population^32^. The current study utilized the FDA-approved Tocris library to identify compounds showing behavioral profiles like calcineurin inhibitors with the final aim of repurposing these drugs for treating Alzheimer’s disease.

## Results

### Development of an automated analysis pipeline for zebrafish behavior

In the current analysis paper, we developed Z-LaP Tracker to quantify zebrafish larval behaviors. Z-LaP Tracker uses modifications of the DeepLabCut framework to monitor zebrafish behavior throughout the experiment phase. DeepLabCut is a markerless pose estimation method based on transfer learning with deep neural networks^17^. It provides a graphical user interface for researchers with minimal programming experience to label key features in a video frame, train a model based on those frames via transfer learning and extract similar features from new experiments. The method has previously been used for pose estimation of videos of adult zebrafish, rodent models, and other animal models^17^. In Z-LaP Tracker, we developed and introduced an additional preprocessing layer using traditional computer vision methods to segregate individual zebrafish on top of the DeepLabCut layer. The necessity of a preprocessing layer arises from the need to detect multiple zebrafish in the same frame. While DeepLabCut does provide multi-animal pose estimation, given that in our experimental setup the zebrafish do not interact with each other it becomes more accurate and reliable to segregate the individual wells before using DeepLabCut to predict zebrafish behaviors.

**Figure 1:**
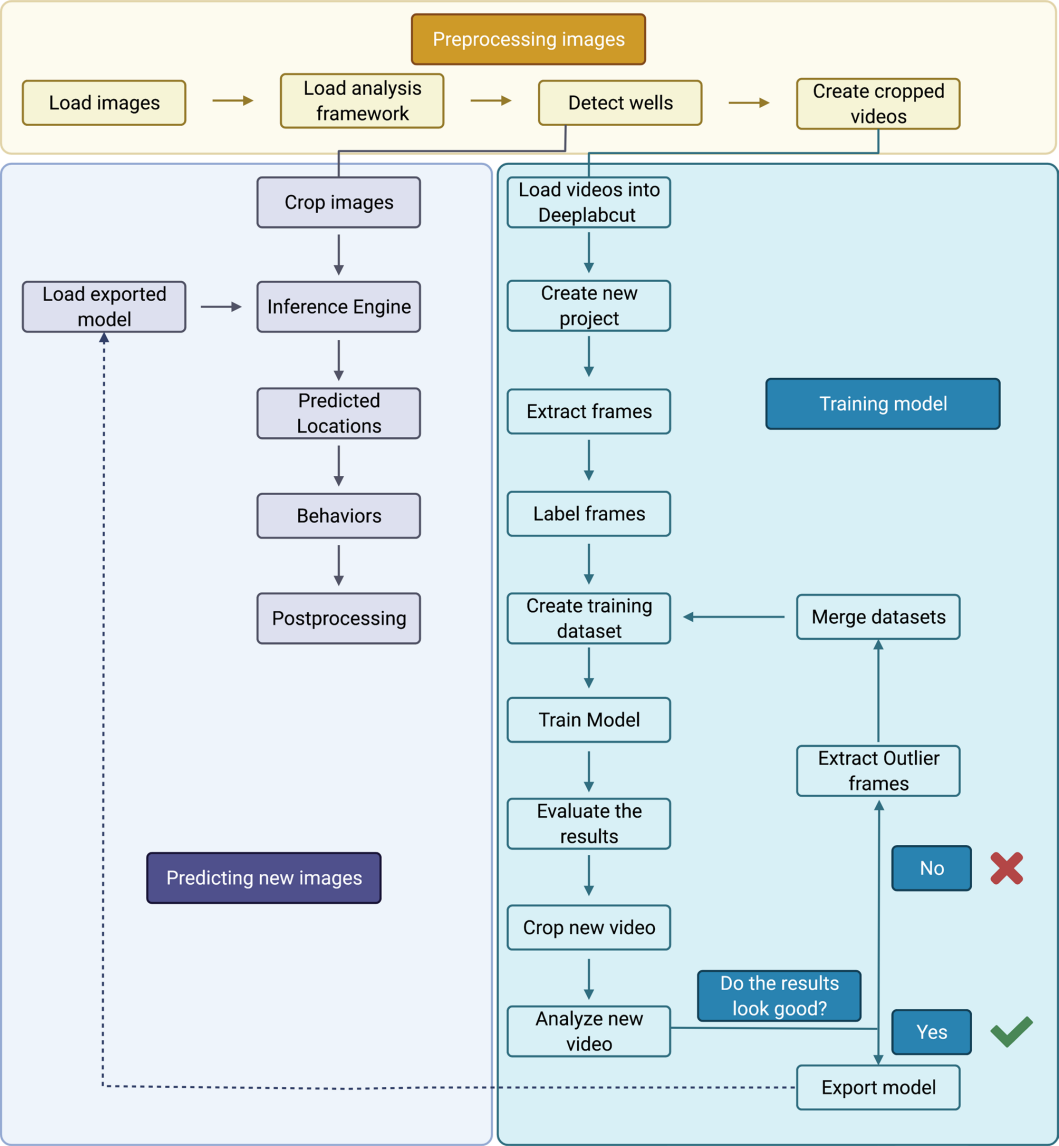
Flowchart for automated behavioral analysis: We implement traditional computer vision methods to segregate individual images, which are then fed into DeepLabCut for training (blue box). The model signature is stored as a protobuf format, which can be utilized for future inference. This pipeline is implemented as python scripts running inside docker containers.

The general workflow is shown in Fig. 1. The first step of our DeepLabCut model training pipeline detects and crops individual wells from images. To achieve this, we utilized the Hough circles function using the Hough Gradient detection method as implemented in OpenCV. For our behavioral images, we used the minimum distance between wells to be at least 150 pixels. The minimum radius and maximum radius were used as runtime parameters for the detection procedure. This implementation gives the Hough Circle routine sufficient freedom to account for any impurities on the plate or any lighting issues when conducting our experiments. Our exact implementation can be found in the source code provided on the GitHub repository (https://github.com/brown-ccv/Automated-Analysis-of-Zebrafish). Having detected the wells as a center coordinate and a radius we crop the image into individual wells (Fig. 1).

To train a deep learning model using DeepLabCut we first determined the zebrafish features that can be used for behavioral analysis. In this regard, there are 2 considerations—(1) the features can be utilized to extrapolate various behaviors utilized in this study, and (2) a trained DeepLabCut model is reliably able to detect these features on previously unseen images. As such, we chose the eyes (left and right) and the yolk of the zebrafish as the features we use for behavioral analysis—we have found that the large contrast between these features and the background makes detection much easier compared to other body parts, for example, the tip of the tail. To train the model we manually annotate the yolk and 2 eyes, using the DeepLabCut interface, in 100 randomly chosen wells and train for ~ 200,000 iterations using the annotated images as training data. Upon completion of the first training round, we bootstrap the model with additional 2 rounds of training (with ~ 50,000 iterations each) by refining labels from the previous round. The model is then exported to a protobuf format to be used in subsequent experimental analysis.

**Figure 2:**
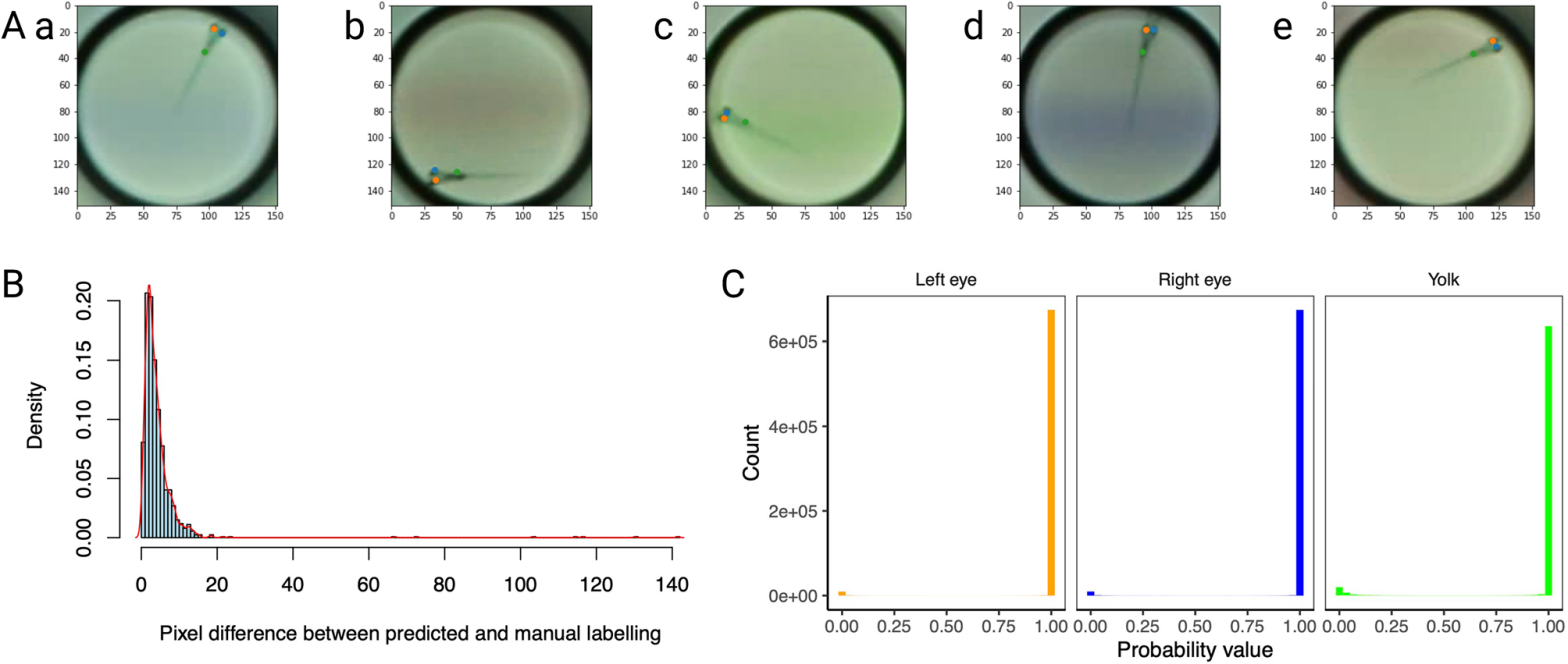
Validation of model: (**A**) The trained DeepLabCut model can detect the left eye (orange dot), right eye (blue dot), and yolk (green dot) across diverse backgrounds such as blank (**Aa**), red moving lines (**Ab**), green moving lines (**Ac**), blue moving lines (**Ad**) and fast red moving lines (**Ae**). (**B**) Euclidean distances between predicted and manually labeled features are described as histograms. The pixel difference between the predicted and manual labeling shifts towards lower pixel difference values after training the model. (**C**) The detection probability of a trained model to accurately detect the left eye (orange bar), right eye (blue bar), and eye (green bar) are shown. The model detects each of the features with very high probability values (= 1).

Before we utilized the trained model for behavioral analysis, we validated the model by checking its accuracy against a validation dataset. The validation dataset consisted of 436 randomly chosen manually labeled previously unseen (a dataset different from the training dataset) images. Figure 2 shows a qualitative and quantitative validation of the model. The Z-LaP tracker can detect specified features (eyes and yolk) across diverse backgrounds (Fig. 2Aa–e). The model accurately predicts the eyes and yolk of zebrafish images with a prediction error of a few (approximately 3–4 pixels) pixels (Fig. 2B). The prediction probabilities for detecting eyes and yolks are high (Fig. 2C) with Z-LaP Tracker. However, the inaccuracies arise when either the image is a blank (black) image, or when the 3 features are not easily distinguishable. Quantitatively this results in a noisy distribution of data. To mitigate these errors, we do not use measurements with a prediction probability (left eye, right eye, and yolk) of < 0.5 for subsequent calculations of behavior.

Lastly, we utilized this model throughout this study to detect zebrafish locations and extrapolate their behaviors. Note that the preprocessing of the experimental images for segregating the wells is identical to the training and inference process. This gives us the ability to reuse code (modules) within the training and inference pipelines. Additionally, model training is done on a single dataset and is sufficiently accurate in subsequent experiments as long as the experimental setup is identical as shown by our validation results.

### Quantification of various novel behavioral parameters for creating a behavioral profile of various drugs

We used the Z-LaP tracker to examine two prior data sets with images of 5-day-old zebrafish larvae. The first data set shows the behavior of zebrafish larvae after exposure to the calcineurin inhibitors cyclosporin (CsA) and tacrolimus^19^. The second data set shows the behavior of zebrafish larvae after exposure to 190 FDA-approved drugs^20^. In both studies, zebrafish larvae (5 dpf) were exposed to various drugs and imaged in four 96-well plates for quantification and analysis of behavior. Using PowerPoint, larvae were subjected to various visual (moving lines) and acoustic stimuli (20 and 1-s interval sound), and images were acquired every 6 s. These images were then analyzed using the automated analysis pipeline optimized for obtaining the position of each larva during the 3-h trial. We then used Excel templates (provided as a supplement) to quantify activity and location. For data processing and extraction of relevant behavioral parameters, we divided 3-h recordings into eighteen 10-min segments. We specifically extracted 25 behavioral parameters to create a behavioral profile of various drugs in zebrafish larvae (Table 1). Zebrafish larvae serve as a valid model for high-throughput behavioral analysis and are known to show a wide range of complex behaviors^7,12,19,20,33,34^. Using the DeepLabCut pipeline, we were able to quantify novel behaviors and subtle changes in them, providing a framework for developing an in-depth behavioral profile for zebrafish larvae exposed to various drugs. Compared to our previous study^19,20^, the current work introduced 15 new behavioral variables. These novel behaviors include measurements of swim speed, larval location in the outer area or edge of the wells, clockwise orientation, upward orientation, and turn angle during various periods of the imaging session (Table 1). The average values of 25 behaviors for all the compounds (calcineurin inhibitors and FDA-approved library) are provided in Supplementary file 1. In comparison to the previous reports^19,20^, the 15 newly added behavioral parameters were specifically helpful to characterize the swimming preference, space utilization, orientation, and turning angles of zebrafish larvae across various stimuli. Addition of these new behaviors aided significantly in the study of the components of complex behaviors observed in larvae exposed to various drugs. For example, one of the newly added behavioral parameters, Sc-1 h describes slow/short swimming movements during the first hour and proved to be significantly higher for 10 μM CsA in addition to the activity during the first hour. This suggests that 10 μM CsA-exposed larvae, in general, show overall increased movement but also exhibit small subtle changes in activity. Similarly, other newly added behavioral variables significantly enhance the sensitivity and quality of behavioral profiles, aiding in the identification of novel drugs showing complex behaviors like CsA.

**Table 1:** Description of the 25 behavioral measures used for hierarchical and K-means cluster analysis.

Name of variable	Name of behavioral parameter	Measure	Relevant period
1 h	1-h activity	Movement (Percent average movement) of larvae during the first hour, i.e., before being exposed to any visual or acoustic stimuli	Period 1 to 6
P15	Period 15 activity	Movement (Percent average movement) of larvae during period 15, i.e., after being exposed to the visual stimuli	Period 15
Hab	Habituation	Change in activity in response to 1-s intervals sound stimuli (first vs. second 5 min of period 17)	Period 17
St	Startle	Change in activity in response to acoustic stimuli at 20 s intervals. The difference in average activity between period 16 and period 15	Period 15 and 16
Ex	Excitability	Change in activity in response to acoustic stimuli at 1 s intervals. The difference in average activity between period 17 and period 16	Period 16 and 17
R	Red	Optomotor response (OMR) using moving red lines. The difference in percent time spent in the upper part of the well between period 8 and period 7	Period 7 and 8
G	Green	Optomotor response (OMR) using moving green lines. The difference in percent time spent in the upper part of the well between period 10 and period 9	Period 9 and 10
B	Blue	Optomotor response (OMR) using moving red lines. The difference in percent time spent in the upper part of the well between period 12 and period 11	Period 11 and 12
FR	Fast red	Optomotor response (OMR) using faster moving red lines. The difference in percent time spent in the upper part of the well between period 14 and period 13	Period 13 and 14
RGB	Red, green, blue	Combined optomotor response (OMR) using moving lines of any color or speed. The average difference in time spent in the upper part of the well between downward-moving line periods and upward-moving line periods	Period 7 to 14
Sc-1 h	Scoot 1 h	Movement (Percent average movement) of larvae in a slow/short swimming (scoot) pattern during the first hour, before being exposed to any visual or acoustic stimuli	Period 1 to 6
Sc-V	Scoot vision	Movement (Percent average movement) of larvae in a slow/short swimming (scoot) pattern during the presentation of moving lines of any color or speed	Period 7 to 14
Bu-1 h	Burst 1 h	Movement (Percent average movement) of larvae in a quick/long swimming (burst) pattern during the first hour, before being exposed to any visual or acoustic stimuli	Period 1 to 6
Bu-V	Burst 1 vision	Movement (Percent average movement) of larvae in a quick/long swimming (burst) pattern during the presentation of moving lines of any color or speed	Period 7 to 14
Ed-1 h	Edge 1 h	Percent time that the larvae are located in the edge of the well (i.e. 50% away from the center) during the first hour	Period 1 to 6
Ed-V	Edge vision	Percent time that the larvae are located in the edge of the well (i.e. 50% away from the center) during periods with moving lines of any color or speed	Period 7 to 14
Cw-1 h	Clockwise 1 h	Percentage of time that the orientation of larvae is clockwise during the first hour	Period 1 to 6
Cw-V	Clockwise vision	Percentage of time that the orientation of larvae is clockwise during periods with moving lines of any color or speed	Period 7 to 14
Or-R	Orientation red	Upward orientation of the larvae while exposed to moving red lines. The difference in percent time facing up between period 8 and period 7. Upward orientation measures whether the larvae are facing in the same direction as the moving lines (positive Orientation value) or not (negative Orientation value)	Period 7 and 8
Or-G	Orientation green	Upward orientation of the larvae while exposed to moving green lines. The difference in percent time facing up between period 10 and period 9	Period 9 and 10
Or-B	Orientation blue	Upward orientation of the larvae while exposed to moving blue lines. The difference in percent time facing up between period 12 and period 11	Period 11 and 12
Or-FR	Orientation fast red	Upward orientation of the larvae while exposed to faster moving red lines. The difference in percent time facing up between period 14 and period 13	Period 13 and 14
Or-RGB	Orientation red, green, blue	Combined upward orientation of the larvae while exposed to moving lines of any color or speed. The average difference in time spent facing up between downward-moving line periods and upward-moving line periods	Period 7 to 14
Turn-1 h	Turn angle 1 h	Change in the larvae’s position angle during the first hour. Turn angle measures whether the larvae have a preference in turn direction	Period 1 to 6
Tabs-1 h	Absolute turn angle 1 h	Absolute change in the larvae’s position angle during the first hour. Absolute turn angle measures how much the larvae turn in either direction	Period 1 to 6

The table describes in detail each of the behavioral measures that were quantified. In particular, the type of measure (for example, absolute value, ratio or percent, and so on) and specific periods of the behavioral test relevant to the quantification of the behavioral measure are also listed.

### Principal components analysis of behavioral parameters

**Figure 3:**
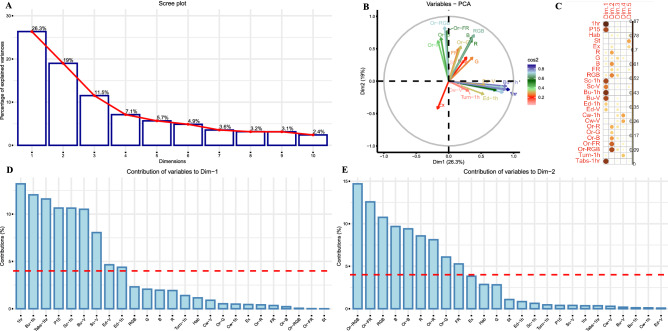
Principal components analysis: the principal component analysis of the dataset identified 22 principal dimensions explaining 100 percent of the variation in the dataset. The scree plot (**A**) shows 10 principal dimensions accounting for 86.74% of the variance. To analyze the contribution of each variable towards the variation in our dataset, we used principal component 1 (Dim1) which explained 26.3% variation, and principal component 2 (Dim2) explaining 19% of the variation (**B**). The correlation plot (**C**) shows the correlation of each variable for the first 5 dimensions of the principal components. The major contributors for Dimension1 (**D**) were the activity-related variables, while Dimension 2 was greatly influenced by the orientation variables (**E**).

We performed principal components analysis on 25 (described in the previous section) behavioral parameters to extract information about the correlation between multivariate variables (Fig. 3). This allowed us to identify a few key principal components (dimensions) explaining most of the variation in the dataset. The principal component analysis of the dataset identified 22 principal dimensions explaining 100 percent of the variation in the dataset. The scree plot (Fig. 3A) shows 10 principal dimensions accounting for 86.74% of the variance. The correlation plot (Fig. 3B) shows the correlation of each variable for the first 5 dimensions of the principal components. To analyze the contribution of each variable towards the variation in our dataset, we used principal component 1 (Dim1) which explained 26.3% variation, and principal component 2 (Dim2) explaining 19% of the variation (Fig. 3C). The variable correlation PCA plot represents the relationship between variables and their contributions to the selected principal components. The variables that are away from the origin have a higher impact on the factor map. For example, 1-h activity and Or-RGB have values near 1 on X- and Y-axes, respectively. The positively correlated variables are grouped on the PCA plot. All the activity parameters clump together on the Dim1 axis while the orientation parameters group together on the Dim2 axis. The negatively correlated variables are grouped in the opposite quadrants on the PCA plot. For example, the orientation variables and excitability show a negative correlation. The major contributors for Dimension1 (Fig. 3D) were the activity-related variables, mainly, activity during the first hour, Burst and scoot activity (during the first hour and vision), Activity during P15, Edge preference, and absolute turn angle during the first hour. Dimension 2 was greatly influenced by the orientation variables, mainly, the upward orientation of larvae during colored moving lines (Or-R, G, B, FR, RGB combined) and optomotor response (R, B, FR).

### K-means clustering and identification of CsA-type drugs

**Figure 4:**
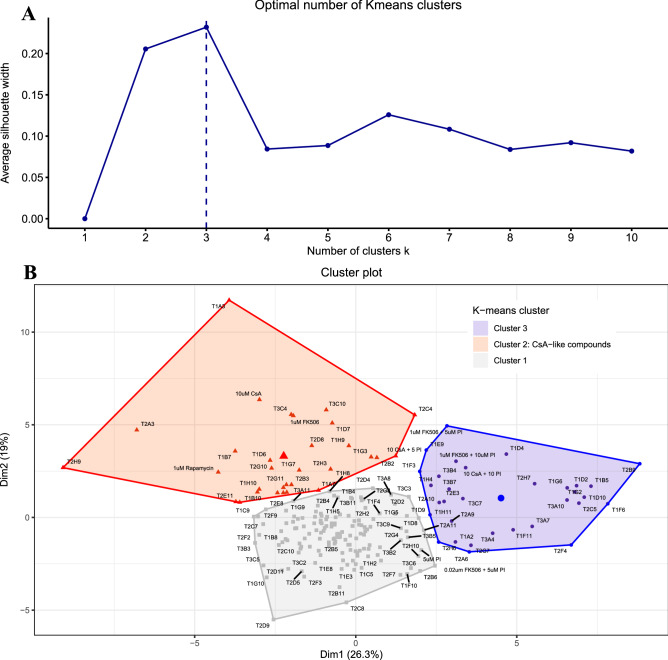
K-means clustering: using the average silhouette width method, we determined that the optimal number of K-means clusters for our dataset was 3 (**A**). We then plotted each of our initial data points on Dim1 (X-axis) and Dim 2 (Y-axis) with the optimal number of clusters (Km = 3) for our dataset. K-means clustering showed in three distinct clusters on the PCA axis (**B**).

We used K-means clustering to identify novel drugs that cluster together with calcineurin inhibitors (CsA and FK506). Using the average silhouette width method, we determined that the optimal number of K-means clusters for our dataset was 3 (Fig. 4A). We then plotted each of our initial data points on Dim1 (X-axis) and Dim 2 (Y-axis) with the optimal number of clusters (Km = 3) for our dataset. K-means clustering showed in three distinct clusters on the PCA axis (Fig. 4B). The K-means cluster contains a cluster means for value for each of the 25 variables in this study (Supplementary file 2). Cluster 1 comprises 141 compounds including the EW and DMSO controls. The average values for Cluster 1 mean were near 0 for most of the behavioral parameters. Cluster 2 contained CsA and FK506 along with 26 other novel compounds. The cluster mean of the CsA-type cluster typically showed high values for activity parameters and low values for orientation variables; a peculiar behavior profile observed for CsA-type drugs. Cluster 3 consisted of 32 compounds including the rescue experimental groups. Cluster 3 means showed values opposite to the CsA-cluster, i.e., lower values for activity parameters and higher values for orientation variables.

### Hierarchical clustering and identification of CsA-type drugs

**Figure 5:**
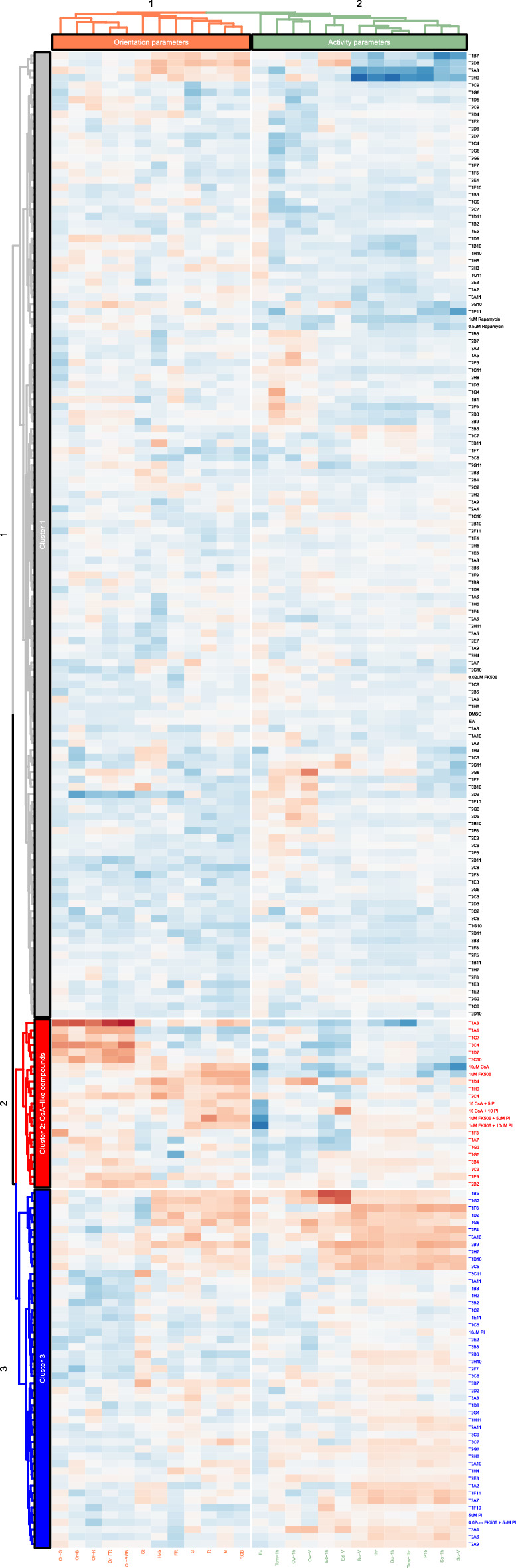
Hierarchical clustering: 25 behavioral parameters were used to produce a heatmap of various behavioral measures for each drug compound. The red-blue color spectrum represents values ranging from − 10 (red) to 5 (blue). Using the hierarchical clustering on the Euclidean distances of each compound, we identified 3 separate clusters depending on the behavioral profile of these drugs. Hierarchical clustering analysis uses the magnitude of effect to reveal clusters of compounds with similar effects on behavior. Cluster 2 (red) was identified and labeled as a CsA-type cluster as CsA and FK506 clustered with 17 novel seemingly unrelated compounds. The CsA-type cluster shows a typical heatmap pattern with lower values for orientation parameters and higher activity parameters. The CsA-type cluster also contained a tight cluster of rescue (CsA/FK506 + PI) experimental groups suggesting that supplementing ProINDY (PI) with CsA/FK506 is not sufficient to rescue behavioral effects produced by calcineurin inhibitors. Cluster 1 (grey) consisted of EW and DMSO controls along with a majority of (132) compounds from the drug library. This cluster did not show larger values for any of the orientation or activity parameters. Cluster 3 (blue) consisted of drugs ProINDY along with 48 other drug compounds. ProINDY activates NFAT via the inhibition of an inhibitor (DYRK1A) and induces behaviors that are nearly opposite to the CsA-induced behaviors. This cluster showed a behavioral profile with larger values orientation and lower activity values; a pattern that was the reverse of CsA-type drugs.

25 behavioral parameters were used to produce a heatmap of various behavioral measures for each drug compound (Fig. 5). The red-blue color spectrum represents values ranging from − 10 (red) to 5 (blue). Using the hierarchical clustering on the Euclidean distances of each compound, we identified 3 separate clusters depending on the behavioral profile of these drugs. Hierarchical clustering analysis uses the magnitude of effect to reveal clusters of compounds with similar effects on behavior. Cluster 2 (red) was identified and labeled as a CsA-type cluster containing CsA and FK506 along with 17 novel other seemingly unrelated compounds. The CsA-type cluster shows a typical heatmap pattern with lower values for orientation parameters and higher activity parameters. The CsA-type cluster also contained a tight cluster of rescue (CsA/FK506 + PI) experimental groups suggesting that supplementing ProINDY (PI) with CsA/FK506 is not sufficient to rescue behavioral effects produced by calcineurin inhibitors. Cluster 1 (gray) consisted of EW and DMSO controls along with a majority of (132) compounds from the drug library. This cluster did not show larger values for any of the orientation or activity parameters. Cluster 3 (blue) consisted of drugs ProINDY along with 48 other drug compounds. ProINDY activates NFAT via the inhibition of an inhibitor (DYRK1A) and induces behaviors that are nearly opposite to the CsA-induced behaviors. This cluster showed a behavioral profile with larger values orientation and lower activity values; a pattern that was the reverse of CsA-type drugs.

### CsA-type drugs and their role in calcineurin signaling

The K-means and hierarchical clustering identified novel compounds targeting a wide range of molecular targets—from kinase inhibitors to adrenergic receptor modulators. The majority (19 out of 32) of these identified compounds were modulators of adrenergic receptors, glucocorticoid receptors, angiotensin receptors, dopamine receptors, and 5-HT receptors while the remaining drugs targeted various enzymes and other receptors. A detailed list of all the identified compounds with their molecular target is described in Table 2. The p values (after Bonferroni adjustment for multiple comparisons) of all the identified compounds for the selected 25 behavioral parameters are listed in Supplementary file 3. In general, a significant effect was observed across all the behavioral variables except startle, vision in green, clockwise movements, and turn angle during the initial hour.

**Table 2:** List of the drugs showing CsA-like behavioral profiles identified using K-means and hierarchical clustering.

Mode of action	Type	Receptor subtype	Compounds	Analysis/clustering method
Adrenergic receptors	Agonist	Non-selective adrenergic alpha receptors	T1A4—dihydroergotamine mesylate	K-means, heatmap
		Adrenergic beta-2 receptors	T2E11—salmeterol xinafoate	K-means
		Adrenergic beta-2 receptors	T2H9—arformoterol tartrate	K-means
	Antagonist	Non-selective adrenergic beta receptors	T1F3—Carvedilol	Heatmap
		Adrenergic alpha-1 receptors	T1A7—prazosin hydrochloride	K-means, heatmap
		Adrenergic alpha-1 receptors	T1G3—doxazosin mesylate	K-means, heatmap
		Adrenergic alpha-1 receptors	T1G7—tamsulosin hydrochloride	K-means, heatmap
		Adrenergic beta-1 receptors	T1H9—nebivolol hydrochloride	K-means, heatmap
Glucocorticoid receptors	N/A	Other	T1B10—dexamethasone	K-means
	Agonist	N/A	T1D5—fluticasone propionate	K-means
	N/A	Other	T3C3—ciclesonide	Heatmap
Angiotensin receptors	Antagonist	Angiotensin AT1 Receptors	T2B3—losartan potassium	K-means
		Angiotensin AT1 Receptors	T2H3—irbesartan	K-means
Dopamine	Agonist	Non-selective Dopamine	T1A3—bromocriptine mesylate	K-means, heatmap
		Non-selective Dopamine	T1E9—cabergoline	Heatmap
		D2 Receptor	T2G11—aripiprazole	K-means
5-HT (serotonin)	Antagonist	Non-selective 5-HT2	T1D6—mirtazapine	K-means
		Non-selective 5-HT	T2A3—asenapine maleate	K-means
	Agonist	5-HT1D receptors	T1H10—sumatriptan succinate	K-means
Estrogen and related receptors	Modulator	N/A	T1B7—tamoxifen citrate	K-means
Vesicular monoamine transporters	Inhibitor	N/A	T1D7—tetrabenazine	K-means, heatmap
Kainate receptors	Antagonist	N/A	T2A2—topiramate	K-means
Cyclooxygenase	Inhibitor	N/A	T2B2—celecoxib	K-means, heatmap
Androgen receptor	Antagonist	N/A	T2C4—flutamide	K-means, heatmap
Pregnane X receptor	Agonist	N/A	T2D8—meclizine dihydrochloride	K-means
VEGFR	Inhibitor	N/A	T2G10—XL 184	K-means
Phosphodiesterases	Inhibitor	N/A	T3B9—tadalafil	K-means
Histone demethylases	Inhibitor	N/A	T3C4—ciclopirox	K-means, heatmap
Raf kinase	Inhibitor	N/A	T3C10—sorafenib	K-means, heatmap
Calcium channels	Blocker	Cav1.x	T1D4—isradipine	Heatmap
EGFR	Inhibitor	N/A	T1G5—iressa	Heatmap
Src kinases	Inhibitor	N/A	T3B4—dasatinib	Heatmap

K-means and heatmap analysis identified a range of drugs known to act on various molecular targets. The table describes in detail the main target receptor type and subtype the drug acts on and its pharmacological mode of action (agonist or antagonist).

## Discussion

Behavioral quantification forms a solid basis for many studies in the field of neuroscience. While zebrafish larvae prove to be an excellent model for genetic studies^5,8^, their use for biobehavioral analysis is limited. With the availability of computational approaches, it is possible nowadays to resolve complex behaviors into simpler bits, which was not possible in the past^16,17^. The behavioral quantitative analysis offers a great way to study animal behavior in the natural environment and/or in response to various stimuli by breaking down a complex set of behaviors into distinct individual elements that can then be interpreted. The current study signifies the importance of developing a machine learning-based method, Z-LaP Tracker, for quantifying novel behavioral parameters. Using Z-LaP Tracker, we quantified 25 behavioral parameters that were used to build a behavioral profile in zebrafish larvae. Furthermore, Z-LaP Tracker can be modified to incorporate various other parameters as needed. One of the most important factors involved in building a comprehensive behavioral profile involves an accurate description of behavior and precise quantification of these behavioral parameters^7^. Moreover, a thorough behavioral profile can only be generated when one considers a variety of behaviors relating to neurological disorders^6^. With the Z-LaP tracker, we were able to pick subtle behavioral patterns and quantify them accurately. The Z-LaP Tracker has greatly influenced how larval behavior can be quantified in high-throughput settings and opened up new avenues for developing deep-learning approaches to study behavior. In our previous studies^19,20^, we used Fiji/Image J macros for detecting zebrafish larvae from the acquired images. An important step in this method involved the selection of regions of interest (ROIs) for each well; a process that was done manually. In Z-LaP Tracker, everything is done in an automated fashion; meaning there is no manual interference involved when detecting larvae. We used the same experimental data and analyzed it using Fiji/ImageJ Vs Z-Lap Tracker, and both methods produced similar values for each of the behavioral parameters (data not shown). The findings from our study suggest that the Z-LaP Tracker and other DepLabCut-based approaches offer an amazing opportunity of studying behavior.

The K-means and hierarchical cluster analysis were performed on the behavioral profile of an FDA-approved drug library to understand the effect of these drugs on the calcineurin-NFAT signaling pathway. The calcineurin-NFAT pathway has been studied in-depth in the activated T-cell of the immune system^35–37^ and is also important for its diverse role in various organ systems, including the nervous system^38–42^. A calcineurin-NFAT signaling pathway is complex and diverse receptors/modulators can act on different components of this signaling pathway to bring out convergent or divergent responses. The K-means cluster analysis identified 29 novel drugs showing behavioral profiles similar to the calcineurin signaling pathway inhibitors while hierarchical clustering identified 17 novel compounds. Using zebrafish Fiji/Image J macros for larvae detection in combination with Hierarchical clustering analysis (using Cluster 3.0), our previous study^20^ had identified 11 compounds that clustered with CsA and FK506. The current study identified a total of 32 novel compounds with CsA-like behavioral profiles using Z-LaP Tracker for the detection of zebrafish larvae in combination with K-means and hierarchical clustering. 9 out of the 11 compounds identified in the previous study also showed up in the CsA-like cluster from the current study and 4 compounds (Nebivolol hydrochloride, Bromocriptine mesylate, Tetrabenazine, and Sorafenib) showed up in the CsA-like cluster using all three clustering methods. In addition to the 9 overlapping compounds, the current study identified additional 22 compounds showing CsA-like behaviors. These results reconfirm the findings from our previous study while also allowing us to detect new novel compounds due to the addition of new behavioral parameters and analysis methods. It is interesting to note that while there was some overlap (11 compounds) in the CsA-like compounds identified using K-means and hierarchical clustering, each clustering method also identified a distinct set of CsA-like drugs that were specific to each clustering method. K-means uses PCA axes for clustering the data; meaning that; it specifically takes into consideration the key behavioral parameters contributing the most towards the variation of the dataset, while hierarchical clustering assigns the same weights for all the behavioral parameters when clustering the data. For example, two of the CsA-like drugs identified were Arformoterol tartrate (using only K-means clustering) and Carvedilol (using only hierarchical clustering) are known to target adrenergic receptors. These two compounds show very distinct behavioral profiles; Arformoterol tartrate clusters with CsA-like compounds mainly because the analysis is based on PCA components but does not show up in hierarchical clustering where each behavioral parameter is given equal weightage.

In our study, we find that both clustering methods hold significant merits and are very valuable for identifying CsA-like compounds.

A schematic of the calcineurin-NFAT pathway with major target receptors identified from our study is summarized in Supplementary file 4. Out of all the identified drugs showing a CsA-like behavioral profile, many drugs are adrenergic agonists or antagonists. Adrenergic receptors are G-protein coupled receptors (GPCRs) known to play roles in both cognitive function and immune function^43–45^. Studies have shown that one of the earliest sites of pathology and degeneration in Alzheimer’s disease is the locus coeruleus (LC), a midbrain region that is known to regulate arousal and is important for learning and memory^46^. The LC is rich in norepinephrine signaling and is known for the regulation of anti-inflammatory activity^47^. The identified adrenergic receptor modulators specifically were agonists of nonselective ɑ (dihydroergotamine mesylate) and β2 receptors (Salmeterol xinafoate, Arformoterol tartrate) and antagonists of nonselective β (Carvedilol), β1 (Nebivolol hydrochloride) and ɑ1 (Prazosin hydrochloride, Doxazosin mesylate, Tamsulosin hydrochloride) receptors. The results of our behavioral analysis suggest that activation of β2 adrenergic receptors and inhibition of ɑ1 receptors might be crucial targets important for developing treatment against neurological disorders like Alzheimer’s disease. Studies in humans and Alzheimer’s mice models have shown contradicting results about the effect of β adrenergic blockers on cognition and physiology^48–53^. The diverse downstream pathways activated by the adrenergic system and the fine balance between these are crucial. In cases where too little or too much signaling occurs, can result in the rapid pathological progression of AD and other neurological diseases^54^. Changes in the adrenergic system are an important early hallmark in the progression of AD^55^. Considering the role of adrenergic receptor activation in inflammation and cognition, the identified adrenergic modulators might help in understanding the crosstalk between calcineurin-pathway-mediated inflammatory and cognitive functions in AD. AD progression occurs mainly in the aging brain. Aging is also a risk factor for other disorders, including hypertension and cardiovascular diseases. Considering the overlapping timeline for AD and hypertension in the aging population^56,57^, it is likely that elderly patients are treated simultaneously for both conditions, making adrenergic modulators a clinically relevant target for repurposing drugs for treating AD.

Other groups of drugs showing CsA-like behavioral profiles were mainly modulators of glucocorticoid receptors (Dexamethasone, Fluticasone propionate, Ciclesonide), angiotensin receptors (Losartan potassium, Irbesartan), 5-HT receptors (Mirtazapine, Asenapine maleate, Sumatriptan succinate), and dopamine receptors (Bromocriptine mesylate, Cabergoline). Glucocorticoid receptors are the ubiquitous low-affinity receptors known to bind to glucocorticoids, a group of steroid hormones that freely cross the blood–brain barrier and are known to play an important role in stress responses, learning, and memory. Previous studies have shown that long-term exposure to high levels of glucocorticoids may be a key risk factor for AD development and progression^58^. In the 3 × Tg-AD mice, a glucocorticoid receptor non-selective antagonist mifepristone, rescued cognitive deficits, reduced Aβ levels and phosphorylation and accumulation of Tau^59^. Our findings also identified angiotensin receptor antagonists (Losartan potassium and Irbesartan) as possible therapeutic drugs for treating AD. Angiotensin receptor activation can lead to the activation various downstream signaling pathways mediated by CaM and PKC leading to transcriptional activation. Our analysis also identified non-selective dopamine agonists (Bromocriptine mesylate and Cabergoline) and serotonin modulators (Mirtazapine, Asenapine maleate, Sumatriptan succinate) which showed a CsA-like behavioral profile. Dopamine and serotonin receptors are GPCRs, similar to other targets described previously, known to act on various downstream signaling pathways resulting in differential transcription of certain genes^54^. A variety of other drug targets identified in the current study are known to act on various other receptors (estrogen, vesicular monoamine transporters, kainate, androgen, Pregnane X, VEGFR, EGFR, Calcium channels) and enzymes (cyclooxygenase, Raf Kinase, Src Kinase, Phosphodiesterases, and Histone demethylases). It is very interesting to observe that such a diverse group of targets can produce a relatively similar behavioral profile. Another fascinating finding is that not all the drugs targeting the same receptor induce a similar behavioral profile. These findings in combination suggest the differential action of various drugs in different organ systems and that many of these drugs might produce their effect through unknown mechanisms.

The etiology and disease progression of Alzheimer’s disorder is complex and many of the drugs that focus specifically on clearing the amyloid β/hyperphosphorylated Tau have failed in clinical trials. One explanation is that the accumulation of amyloid β/hyperphosphorylated Tau does not correlate well with cognitive decline, a hallmark of late AD^60,61^. It is, therefore, possible that finding the drugs that target molecular mechanisms involved in early disease progression might prove to be successful in developing therapeutics for treating Alzheimer's disease. Considering the complexity of Alzheimer’s disease etiology and elusive molecular targets, drug repurposing might be one of the most cost/time-effective strategies for developing a treatment for Alzheimer's disease. An interesting study supporting the role of the calcineurin signaling pathway in Alzheimer's is from a human population study where transplant patients chronically treated with calcineurin inhibitors have a significantly lower incidence of AD/dementia as compared to the general population. The current study identified novel drugs showing a CsA-like profile suggesting that these targets might have a novel role in Alzheimer’s disease progression.

## Materials and methods

All the materials and methods for conducting behavioral experiments were identical to the methods used in our earlier study^20^ and are described briefly in this section. The current study applied a new method for tracking zebrafish larvae and data analysis on an existing behavioral dataset^20^.

### Experimental animals

All experiments carried out in the current study were following federal regulations and guidelines for the ethical and humane use of animals and have been approved by Brown University’s Institutional Animal Care and Use Committee (IACUC). All the experiments were carried out using 5 days post fertilization (dpf) larvae obtained from adult wild-type zebrafish (*Danio rerio*). Adult zebrafish are maintained in the Animal Care Facility at Brown University. The existing adult wild-type zebrafish line in the facility is a genetically diverse outbred strain with a mixed population of males and females. The zebrafish are maintained on a 14-h light, 10-h dark cycle in a Marineland Vertical Aquatic Holding System. The zebrafish adults are fed daily with Gemma Micro 300 and frozen brine shrimp during the light cycle. Collection of zebrafish embryos and their growth to (5 dpf) larval stages was achieved using previously described methods^19,20,33,34^. Zebrafish embryos from 0 to 5 dpf were maintained at 28.5 °C in 2L culture trays with egg water (60 mg/L sea salt (Instant Ocean) and 0.25 mg/L methylene blue in deionized water (pH 7.2). Embryos and larvae in 2L culture trays are maintained on a 12 h light/12 h dark cycle. Zebrafish use complex polygenic features for sex determination^62^. Both males and females have juvenile ovaries between 2.5 and 4 weeks of development making it impossible to determine the sex of embryos and larvae at early stages^62^. At 5 dpf, zebrafish larvae (approximately 4 mm in size) display various complex behaviors and do not need an external food source as they consume nutrients available in the yolk sac^63^. After the behavioral experiments were done, the 5 dpf zebrafish larvae were euthanized by rapid chilling followed by immersing them in the bleach solution (1 part bleach to 5 parts tank water) for 15 min.

### Experimental design

Treatment groups and controls were imaged on the same day and repeat experiments were carried out on different days. Sample size (n = 48 per treatment group) was determined a priori, based on analyses of behavior in prior studies^19,20,33,34,63,64^. For each of the treatment groups, larvae were obtained from at least 3 independent clutches. For every imaging experiment, in addition to the treatment groups, egg water, and DMSO controls were run. To avoid biases, the placement of plates and loading of 5-dpf zebrafish larvae in 384-wells were conducted in random order. Behavioral analysis is sensitive to circadian fluctuations^65^ and so, imaging was performed in the 1–5 pm time window. With many treatment groups and using automated methods for imaging, image analysis, and data processing; we were able to get robust reliable behavioral measures. The experimental design, the sample size of zebrafish larvae, and the statistical analysis reported in the current study are in accordance with the ARRIVE (Animal Research: Reporting of In Vivo Experiments) guidelines.

### Pharmacological treatments

Using 96-well ProxiPlates (PerkinElmer, 6006290), 5 dpf zebrafish larvae were incubated in treatment solutions for 3 h before imaging, and for 3 h during the imaging session. Zebrafish larvae were treated with 190 FDA-approved compounds using a Tocris small-molecule library (Tocris Bioscience, Cat. No. 7200). The library contained 10 mM stocks dissolved in dimethyl sulfoxide (DMSO), which was diluted 1000 × in egg water to a 10 µM final concentration. The control groups consisted of untreated larvae in egg water and larvae treated with 1 µl/ml DMSO serving as vehicle control. The behavioral effects of FDA-approved drugs were compared to previously obtained results^20^ with 10 µM cyclosporine A (CsA, Enzo Life Sciences), 1 µM tacrolimus (FK506, Enzo Life Sciences), 1 µM rapamycin (Santa Cruz Biotechnology), 5 and 10 µM proINDY (Tocris Bioscience) and a rescue experimental group typically consisting of calcineurin inhibitor along with proINDY. The rescue group for the current study consisted of four combinations—10 µM CsA + 5 μM proINDY, 10 µM CsA + 10 μM proINDY, 1 µM tacrolimus + 5 μM proINDY and 1 µM tacrolimus + 10 μM proINDY.

### Imaging setup

**Figure 6:**
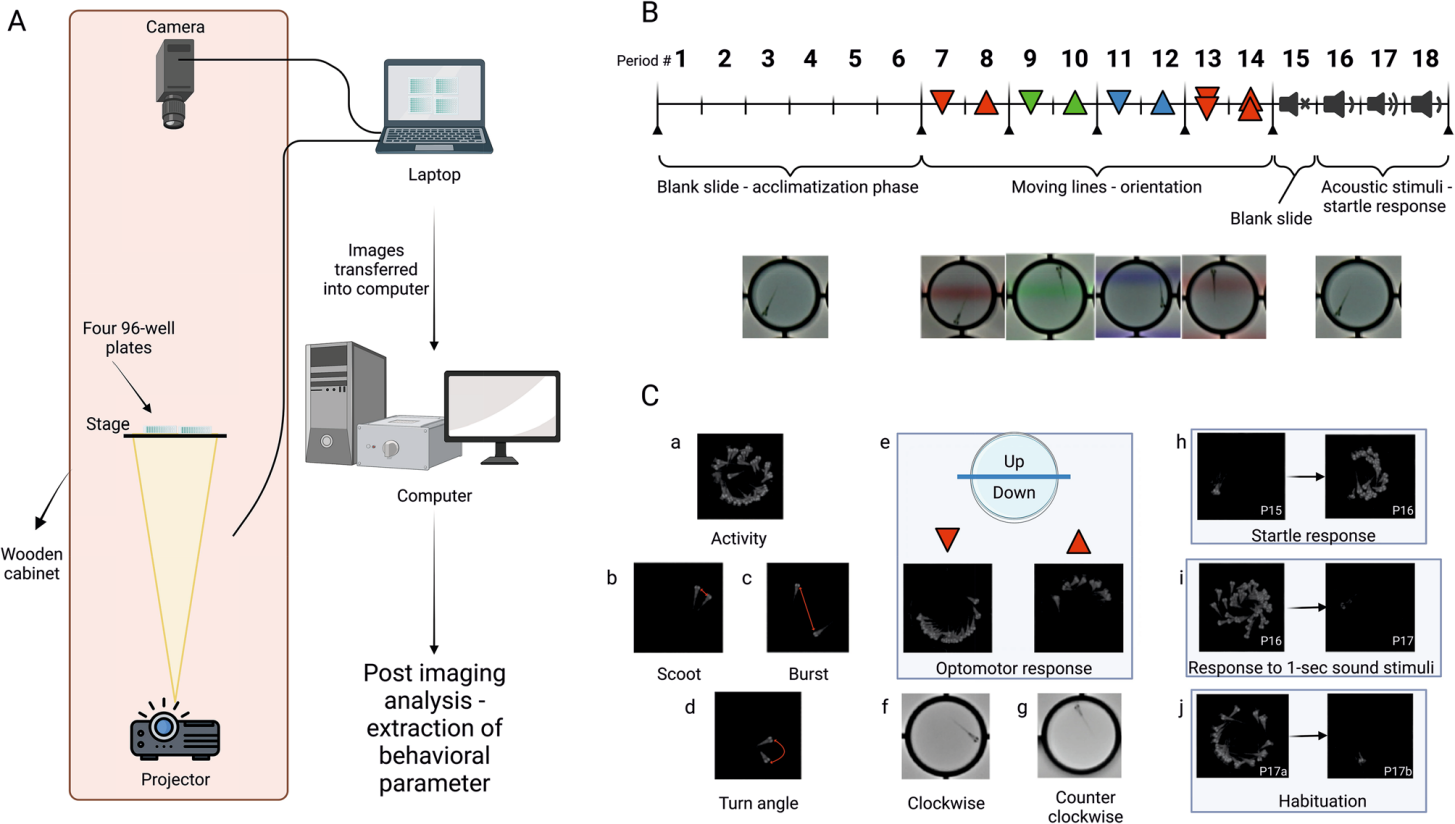
Experimental setup, timeline and description of behavioral variables: (**A**) experimental setup: the imaging setup in the laboratory consists of a projector attached to a laptop using PowerPoint for presenting visual/acoustic stimuli, a staging area (which can hold up to four 96-well plates), and a high-resolution overhead camera that clicks photos of 96-well plates at a set frequency (10 images per minute). The data is analyzed post-imaging using an automated python pipeline based on DeepLabCut. (**B**) Timeline for the presentation of visual and acoustic stimuli: during the first hour, a blank slide was presented, followed by moving lines of red, green, blue, and fast green in an up/down direction, followed by acoustic stimuli of 20-s and 1-s intervals. (**C**) 25 behavioral parameters were quantified for behavioral profiling: some example behaviors analyzed are shown in (**Ca**) activity during the first hour; (**Cb**) scoot slow/short swimming; (**Cc**) burst quick/long swimming; (**Cd**) absolute turn angle; (**Ce**) optomotor quick/long response—movement in the direction of moving lines; (**Cf**) clockwise; (**Cg**) counterclockwise swimming; (**Ch**) startle response—sudden activity in response to an auditory stimulus; (**Ci**) less excitability in response to 1-s interval sound stimulus and (**Cj**) habituation—decrease in activity upon presentation of the repeated sound stimulus.

The imaging system for the automated behavioral analysis holds four 96-well plates for 384-well high throughput behavioral profiling. The imaging setup consists of a high-resolution camera, transparent stage, projector, and a laptop computer (Fig. 6A). The camera, stage for holding plates, and M5 LED pico projector (Aaxa Technologies) with a 900 lumens LED light source are placed in a wooden cabinet maintained at 28.5 °C. Wooden cabinet helps to maintain larvae in optimum environmental condition and reduces external artifacts (changes in ambient light and dampens external sound). The high-resolution camera (18-megapixel Canon EOS Rebel T6 with an EF-S 55–250 mm f/4.0–5.6 IS zoom lens) is set to take pictures every 6 s for a total of 3 h. Based on our prior studies^19,20,33,34^, acquiring images every 6 s allowed us to capture selected relevant behaviors with great detail while also maintaining enough storage/processing for longer periods of experiments A transparent stage is used to hold up to four 96-well plates. The inner diameter of a well is 7.15 mm, which corresponds to 150 pixels in the image (48 μm per pixel). The camera is connected to a continuous power supply (Canon ACK-E10 AC Adapter) and controlled by a laptop computer using Canon’s Remote Capture software (EOS Utility, version 3 included with the camera). Two small speakers (OfficeTec USB Computer Speakers Compact 2.0 System) are used to provide sound stimulus and are connected by USB to the laptop computer and set to maximum volume (85 dBA). The projector is used for background illumination and the display of visual stimuli using Microsoft PowerPoint. The camera settings used for the current study were: ISO200, Fluorescent, F5, and 1/5 exposure. These settings worked the best for obtaining optimal color separation in the automated image analysis.

### Behavioral assay

The behavioral assay used in the current study was identical to the previous study^19,20,33,34^ conducted in the lab. In short, a 3-h long PowerPoint presentation was used to deliver visual and acoustic stimuli to larvae. The exact timeline of each stimulus is shown in Fig. 6B. The initial hour of the trial was considered the acclimatization phase during which larvae did not receive any visual/auditory stimuli (blank slide). The behaviors recorded during this period were used as a measure of baseline activity without any external stimuli. The 1-h blank slide was followed by 80 min of visual stimuli (moving lines of red, green, blue, and fast red in un/down orientation), a 10-min period without visual or acoustic stimuli, and 30 min with acoustic stimuli (20-s interval sound pulse and 1-s interval sound pulse). Earlier studies have shown that zebrafish larvae will show an optomotor response or OMR (i.e., they swim in the same direction as moving lines) in response to moving lines^33,66^. The sequence, timing, and details of the PowerPoint slide background (Supplementary file 5) were identical to the previous study^20^. Using automated image analysis and subsequent data processing (described in the sections below) we quantified various behaviors (some of them are described in Fig. 6C) from different periods of the experiment.

### Automated image analysis

We developed an automated image processing framework Z-LaP Tracker, a modification of DeepLabCut (DLC), to detect zebrafish locations and orientations in images. We used a high-performance computing cluster; OSCAR (Ocean State Center for Advanced Resources) for analyzing zebrafish behavior. OSCAR provides large-scale computing resources (GPUs-NVIDIA V100) which greatly elevates the extent of computational analysis that can be done in a shorter time compared to other traditional computers. OSCAR allows the automated analysis for zebrafish behavior to be completed in 30 min while the same analysis takes approximately 3 h using a traditional computer (Processor Intel [R] Xeon [R] CPU E5-2643 v4 @ 3.40 GHz RAM 128 GB). All the details about the information, usage and installation of the Z-LaP Tracker can be found on the GitHub repository (https://github.com/brown-ccv/Automated-Analysis-of-Zebrafish). The imaging analysis automatically detects zebrafish eyes and yolk using deep learning models. This image-processing pipeline can detect zebrafish larvae in four 96-well plates with multiple treatment groups. The main steps involved in automated image analysis were preprocessing images, training the model, and predicting new images (in diverse backgrounds). The output file of the automated image analysis contains approximately 22.8 million data points, i.e. 33 columns with information on the image, well, larval movement, larval location, larval orientation, and detection probabilities of each feature (left eye, right eye, and yolk) and 691,200 rows showing this information for each well in subsequent images (384 wells × 1800 images). The output file (Supplementary file 6) includes the image number, imaging period (18 × 10-min periods), well number, and XY coordinates of the yolk. These XY coordinates are used to calculate if a larva moved (> 3 pixels in 6 s) and if a larva is in the upper half of a well (in a horizontal plane). These basic measures are similar to measures of behavior obtained by automated image analysis with ImageJ^20^. Z-LaP Tracker provides additional information, beyond these basic measures. Specifically, it provides measures of Speed, Scoot, Burst, Burst-Up, Edge, % Edge, CW, Angle, Upw, Turn, Tabs, XY coordinates of the left eye, XY coordinates of the right eye, and the probabilities of correct recognition of the yolk, left eye and right eye. The description of behavioral parameters and computation method is described in detail in Supplementary file 6. The output file from the Z-LaP Tracker analysis was then used to quantify relevant zebrafish behaviors (described in the section below).

### Data processing

The output files are processed using two MS Excel templates (A and B—included in the supplementary information—Supplementary files 7 and 8 respectively). Template A calculates 25 behaviors for each larva in the 384-well experiments (Table 1). The definition and description for quantification of each behavior are as follows: (1) move: percentage of time that a larva moves (% move), (2) up: percentage of time that a larva is located in the upper half of the well (% up), (3) speed: average speed of each larva, (4) scoot: percentage of time that a larva performs smaller movements (between 3 and 20 pixels), (5) burst: percentage of time that a larva performs bigger movements (above 20 pixels), (6) B-Up: percentage of time that larva displays burst activity in the upper half of well, (7) absolute and percent edge: percentage of time that larva spent around the edge of the well, (8) clockwise: percentage of time that larva was observed in clockwise orientation, (9) angle: orientation of larval body axis in relation to the horizontal axis of the well (− 180° to + 180°), (10) Upw: upward orientation of larva, (11) turn: change in the orientation between subsequent images, (12) Tabs: absolute turn angle. To measure the optomotor response (OMR), larval locations are compared between two 10-min periods when visual stimuli move up vs. down. The criteria for the exclusion of certain data points were set a priori in Excel template A. Data points in which the detection probability of the left eye, right eye, or yolk is less than 0.5 were excluded. In addition, the template automatically excludes zebrafish larvae that move less than 1% of the time in a 3-h recording. Also, larvae that move less than 5% of the time in a 10-min period are automatically excluded from OMR measurements during that period. In Template A, the primary outcome measures are summarized for each experimental group in the ‘Summary’ tab. Template B combines the summary sheets of multiple experiments. This template calculates the average values per treatment group and calculates the differences between these groups as compared to the DMSO vehicle controls. These differences are expressed in percentage points (% points). The template tests differences between DMSO controls and treated groups for statistical significance and displays color-coded behavioral profiles for easier behavioral pattern visualization.

### Principal components and cluster analyses

The changes in 25 behavioral measures (described in earlier sections—Table 1) were compared to the DMSO vehicle controls. The behavioral profiles were created in MS Excel (template A—provided as supplementary file 7) and then using MS Excel (template B—provided as supplementary file 8), the average values of the treated groups (n = 48 larvae per drug) were calculated. Subsequently, the average values of the DMSO vehicle controls (n = 844 larvae) were subtracted from each of the treatment group values to standardize the data set. These values were then used for principal components analysis, K-means clustering, and hierarchical clustering analysis using R (R Studio version 4.2.0). For data analysis in R, we used the following packages: ‘ggplot2’, ‘dplyr’, ‘tidyr’, ‘FactoMineR’, ‘factoextra’, ‘corrplot’, ‘cluster’, and ‘dendextend’. Each of the 25 behavioral parameters was assigned equal weightage for clustering analyses. Also, we used the Euclidian distance similarity metric with Ward’s minimum variance linkage for K-means and hierarchical clustering.

### Statistical analysis

All the statistical tests and data visualization were conducted using Microsoft Excel 2016 and/or R statistical language. The values of our data set were not normally distributed, had unequal sample sizes per experimental group, and had unequal between-group variance. Due to these conditions, a non-parametric Welch’s unequal variance t-test was used. To correct for multiple comparisons, a Bonferroni correction was applied. In the Tocris screen, we compared 190 drugs to the DMSO vehicle controls and differences were considered significant when p < 2.6 × 10^−4^ (0.05/190), p < 5.3 × 10^−5^ (0.01/190), or p < 5.3 × 10^−6^ (0.001/190). This conservative approach was important particularly in high-throughput large datasets to decrease the chance of false positives.

## Supplementary Information

Supplementary Information 1.

Supplementary Information 2.

Supplementary Information 3.

Supplementary Information 4.

Supplementary Information 5.

Supplementary Information 6.

Supplementary Information 7.

Supplementary Information 8.

## Data Availability

Data, instructions for installation/usage of the Z-LaP Tracker image analysis pipeline, codes, and the materials used in the analysis are available in the supplementary information.
