# Supplementary figures and images for: Zebrafish Larvae Position Tracker (Z-LaP Tracker): a high-throughput deep-learning behavioral approach for the identification of calcineurin pathway-modulating drugs using zebrafish larvae
Source: Sci Rep. 2023 Feb 23;13:3174. doi: 10.1038/s41598-023-30303-w (PMC9950053; doi:10.1038/s41598-023-30303-w)

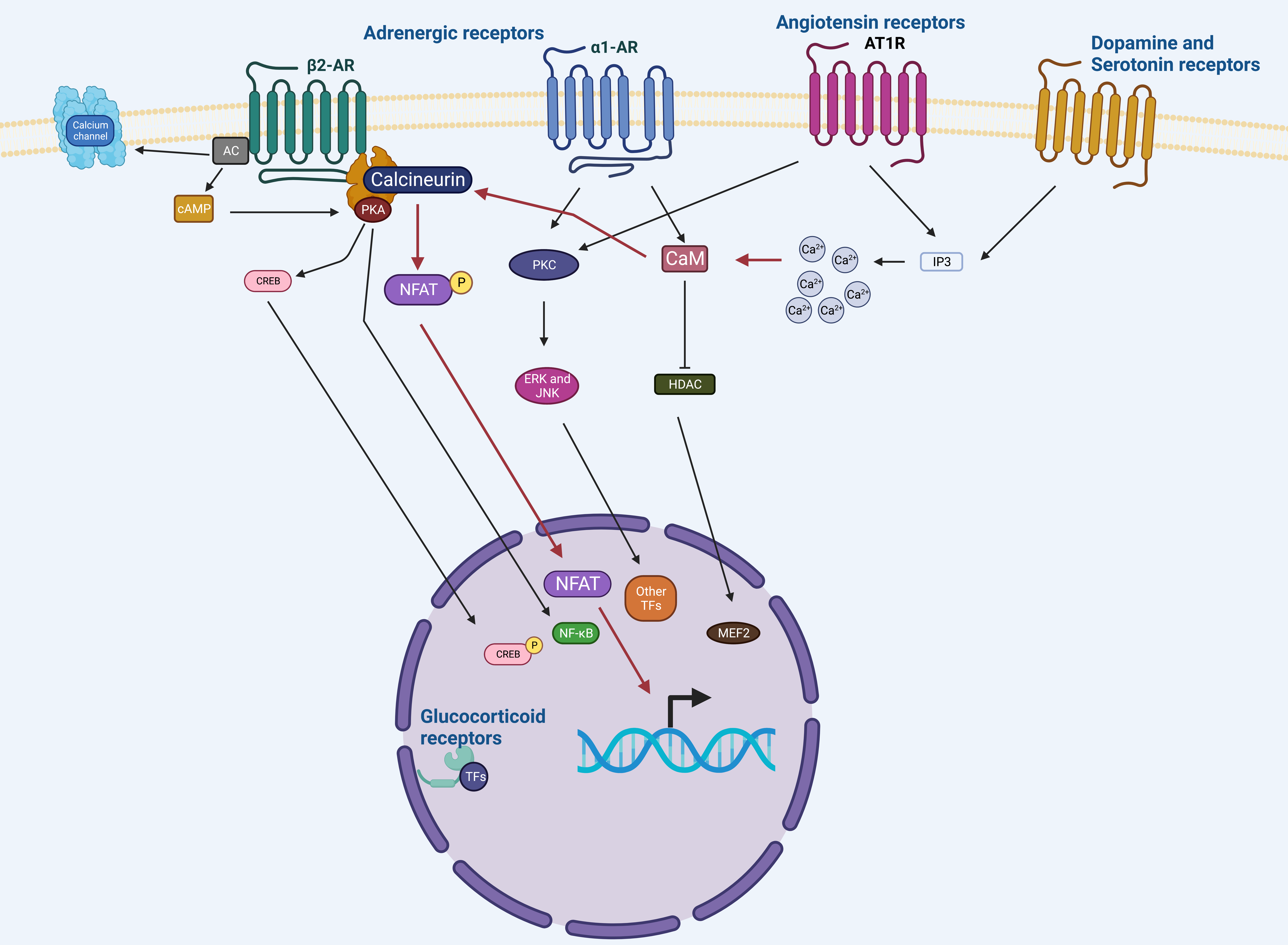

Supplement: Supplementary file 4 — Supplementary Information 4. [file 41598_2023_30303_MOESM4_ESM.png]
