# Supplementary material for: Zebrafish Larvae Position Tracker (Z-LaP Tracker): a high-throughput deep-learning behavioral approach for the identification of calcineurin pathway-modulating drugs using zebrafish larvae
Source: Sci Rep. 2023 Feb 23;13:3174. doi: 10.1038/s41598-023-30303-w (PMC9950053; doi:10.1038/s41598-023-30303-w)

## Slide 1
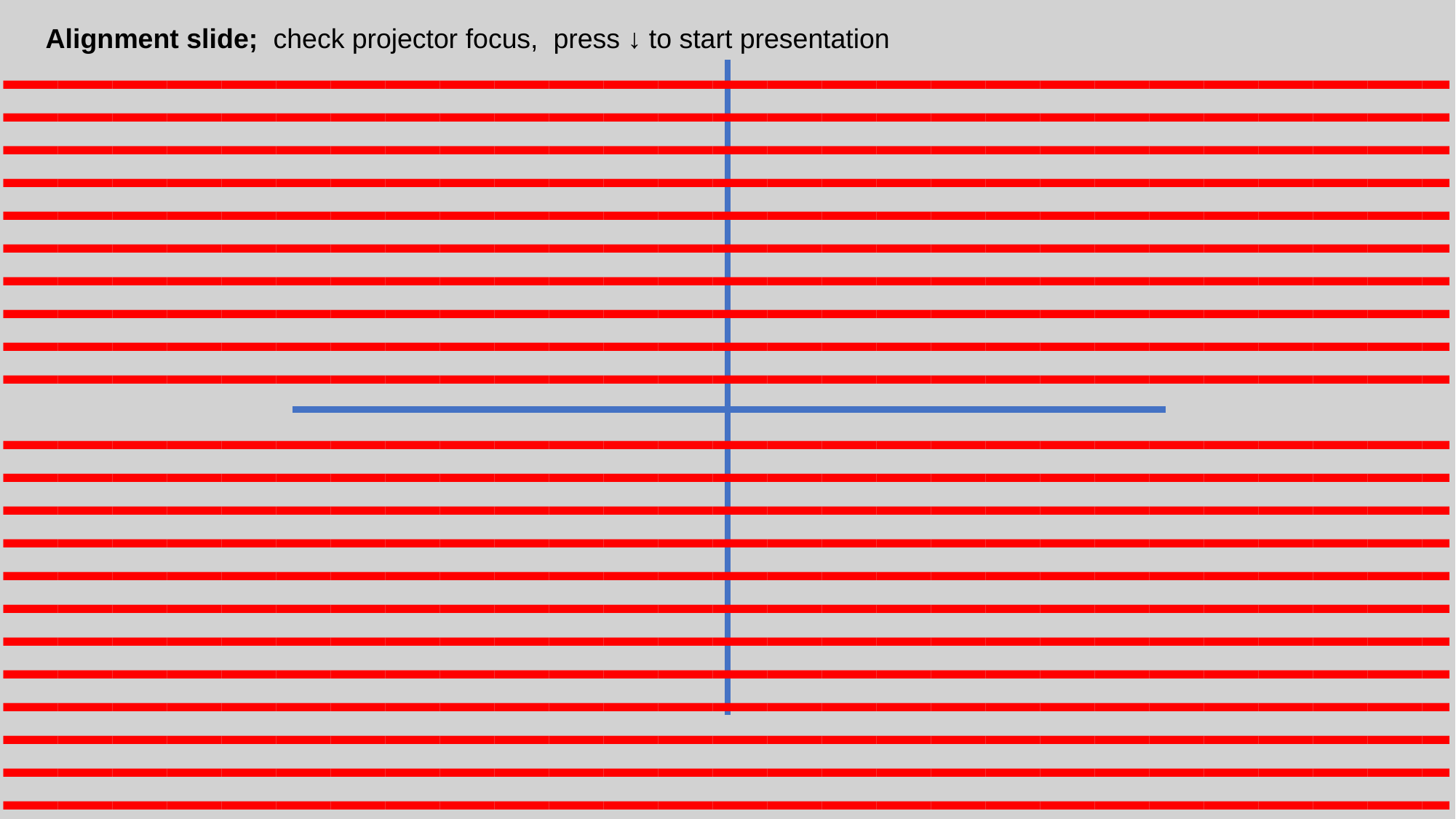

Alignment slide; check projector focus, press ↓ to start presentation
▬▬▬▬▬▬▬▬▬▬▬▬▬▬▬▬▬▬▬▬▬▬▬▬▬▬▬▬▬▬▬▬▬▬▬▬▬▬▬▬▬▬▬▬▬▬▬▬▬▬▬▬▬
▬▬▬▬▬▬▬▬▬▬▬▬▬▬▬▬▬▬▬▬▬▬▬▬▬▬▬▬▬▬▬▬▬▬▬▬▬▬▬▬▬▬▬▬▬▬▬▬▬▬▬▬▬
▬▬▬▬▬▬▬▬▬▬▬▬▬▬▬▬▬▬▬▬▬▬▬▬▬▬▬▬▬▬▬▬▬▬▬▬▬▬▬▬▬▬▬▬▬▬▬▬▬▬▬▬▬
▬▬▬▬▬▬▬▬▬▬▬▬▬▬▬▬▬▬▬▬▬▬▬▬▬▬▬▬▬▬▬▬▬▬▬▬▬▬▬▬▬▬▬▬▬▬▬▬▬▬▬▬▬
▬▬▬▬▬▬▬▬▬▬▬▬▬▬▬▬▬▬▬▬▬▬▬▬▬▬▬▬▬▬▬▬▬▬▬▬▬▬▬▬▬▬▬▬▬▬▬▬▬▬▬▬▬
▬▬▬▬▬▬▬▬▬▬▬▬▬▬▬▬▬▬▬▬▬▬▬▬▬▬▬▬▬▬▬▬▬▬▬▬▬▬▬▬▬▬▬▬▬▬▬▬▬▬▬▬▬
▬▬▬▬▬▬▬▬▬▬▬▬▬▬▬▬▬▬▬▬▬▬▬▬▬▬▬▬▬▬▬▬▬▬▬▬▬▬▬▬▬▬▬▬▬▬▬▬▬▬▬▬▬
▬▬▬▬▬▬▬▬▬▬▬▬▬▬▬▬▬▬▬▬▬▬▬▬▬▬▬▬▬▬▬▬▬▬▬▬▬▬▬▬▬▬▬▬▬▬▬▬▬▬▬▬▬
▬▬▬▬▬▬▬▬▬▬▬▬▬▬▬▬▬▬▬▬▬▬▬▬▬▬▬▬▬▬▬▬▬▬▬▬▬▬▬▬▬▬▬▬▬▬▬▬▬▬▬▬▬
▬▬▬▬▬▬▬▬▬▬▬▬▬▬▬▬▬▬▬▬▬▬▬▬▬▬▬▬▬▬▬▬▬▬▬▬▬▬▬▬▬▬▬▬▬▬▬▬▬▬▬▬▬
▬▬▬▬▬▬▬▬▬▬▬▬▬▬▬▬▬▬▬▬▬▬▬▬▬▬▬▬▬▬▬▬▬▬▬▬▬▬▬▬▬▬▬▬▬▬▬▬▬▬▬▬▬
▬▬▬▬▬▬▬▬▬▬▬▬▬▬▬▬▬▬▬▬▬▬▬▬▬▬▬▬▬▬▬▬▬▬▬▬▬▬▬▬▬▬▬▬▬▬▬▬▬▬▬▬▬
▬▬▬▬▬▬▬▬▬▬▬▬▬▬▬▬▬▬▬▬▬▬▬▬▬▬▬▬▬▬▬▬▬▬▬▬▬▬▬▬▬▬▬▬▬▬▬▬▬▬▬▬▬
▬▬▬▬▬▬▬▬▬▬▬▬▬▬▬▬▬▬▬▬▬▬▬▬▬▬▬▬▬▬▬▬▬▬▬▬▬▬▬▬▬▬▬▬▬▬▬▬▬▬▬▬▬
▬▬▬▬▬▬▬▬▬▬▬▬▬▬▬▬▬▬▬▬▬▬▬▬▬▬▬▬▬▬▬▬▬▬▬▬▬▬▬▬▬▬▬▬▬▬▬▬▬▬▬▬▬
▬▬▬▬▬▬▬▬▬▬▬▬▬▬▬▬▬▬▬▬▬▬▬▬▬▬▬▬▬▬▬▬▬▬▬▬▬▬▬▬▬▬▬▬▬▬▬▬▬▬▬▬▬
▬▬▬▬▬▬▬▬▬▬▬▬▬▬▬▬▬▬▬▬▬▬▬▬▬▬▬▬▬▬▬▬▬▬▬▬▬▬▬▬▬▬▬▬▬▬▬▬▬▬▬▬▬
▬▬▬▬▬▬▬▬▬▬▬▬▬▬▬▬▬▬▬▬▬▬▬▬▬▬▬▬▬▬▬▬▬▬▬▬▬▬▬▬▬▬▬▬▬▬▬▬▬▬▬▬▬
▬▬▬▬▬▬▬▬▬▬▬▬▬▬▬▬▬▬▬▬▬▬▬▬▬▬▬▬▬▬▬▬▬▬▬▬▬▬▬▬▬▬▬▬▬▬▬▬▬▬▬▬▬
▬▬▬▬▬▬▬▬▬▬▬▬▬▬▬▬▬▬▬▬▬▬▬▬▬▬▬▬▬▬▬▬▬▬▬▬▬▬▬▬▬▬▬▬▬▬▬▬▬▬▬▬▬
▬▬▬▬▬▬▬▬▬▬▬▬▬▬▬▬▬▬▬▬▬▬▬▬▬▬▬▬▬▬▬▬▬▬▬▬▬▬▬▬▬▬▬▬▬▬▬▬▬▬▬▬▬
▬▬▬▬▬▬▬▬▬▬▬▬▬▬▬▬▬▬▬▬▬▬▬▬▬▬▬▬▬▬▬▬▬▬▬▬▬▬▬▬▬▬▬▬▬▬▬▬▬▬▬▬▬

## Slide 2
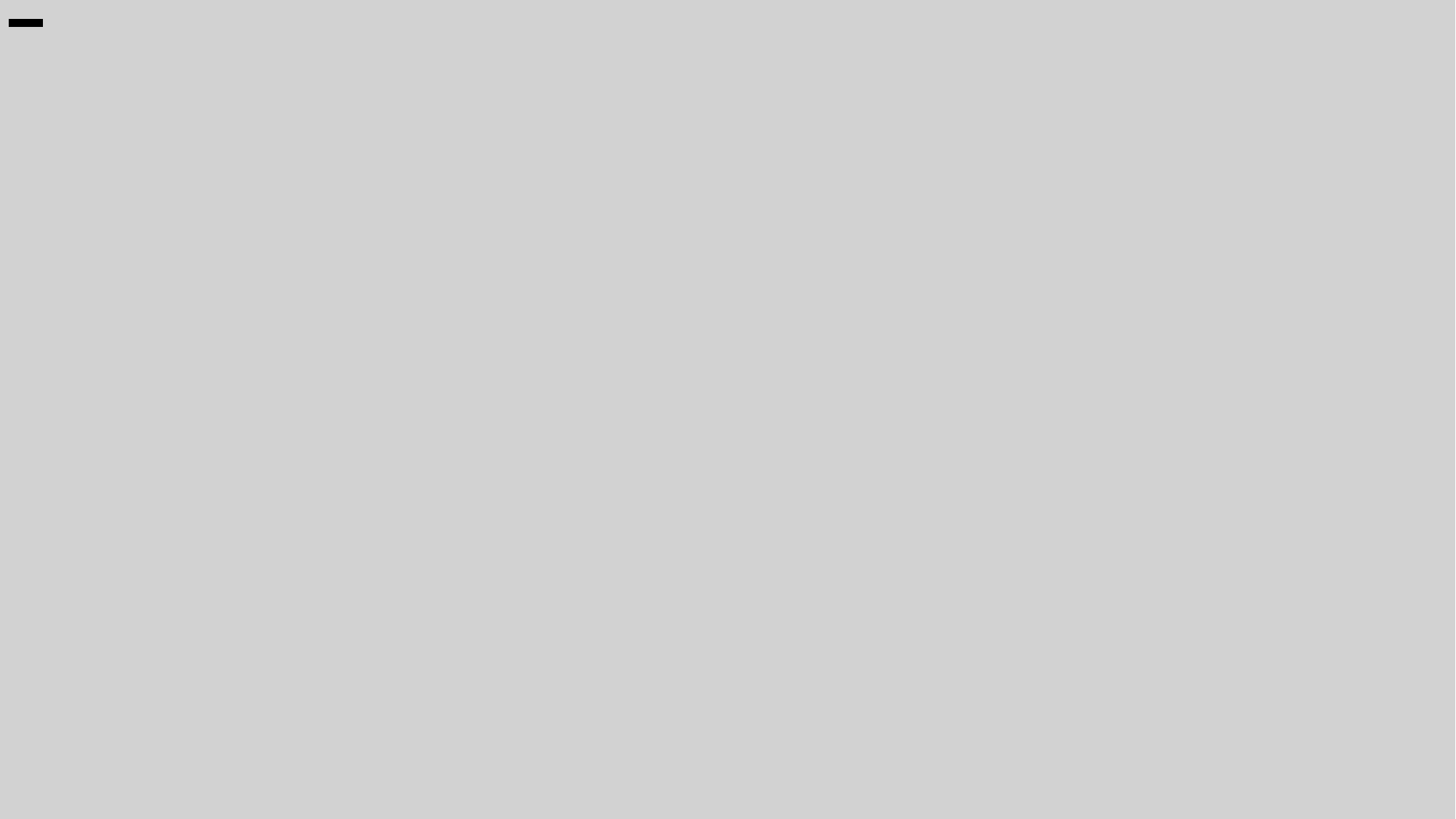

## Slide 3
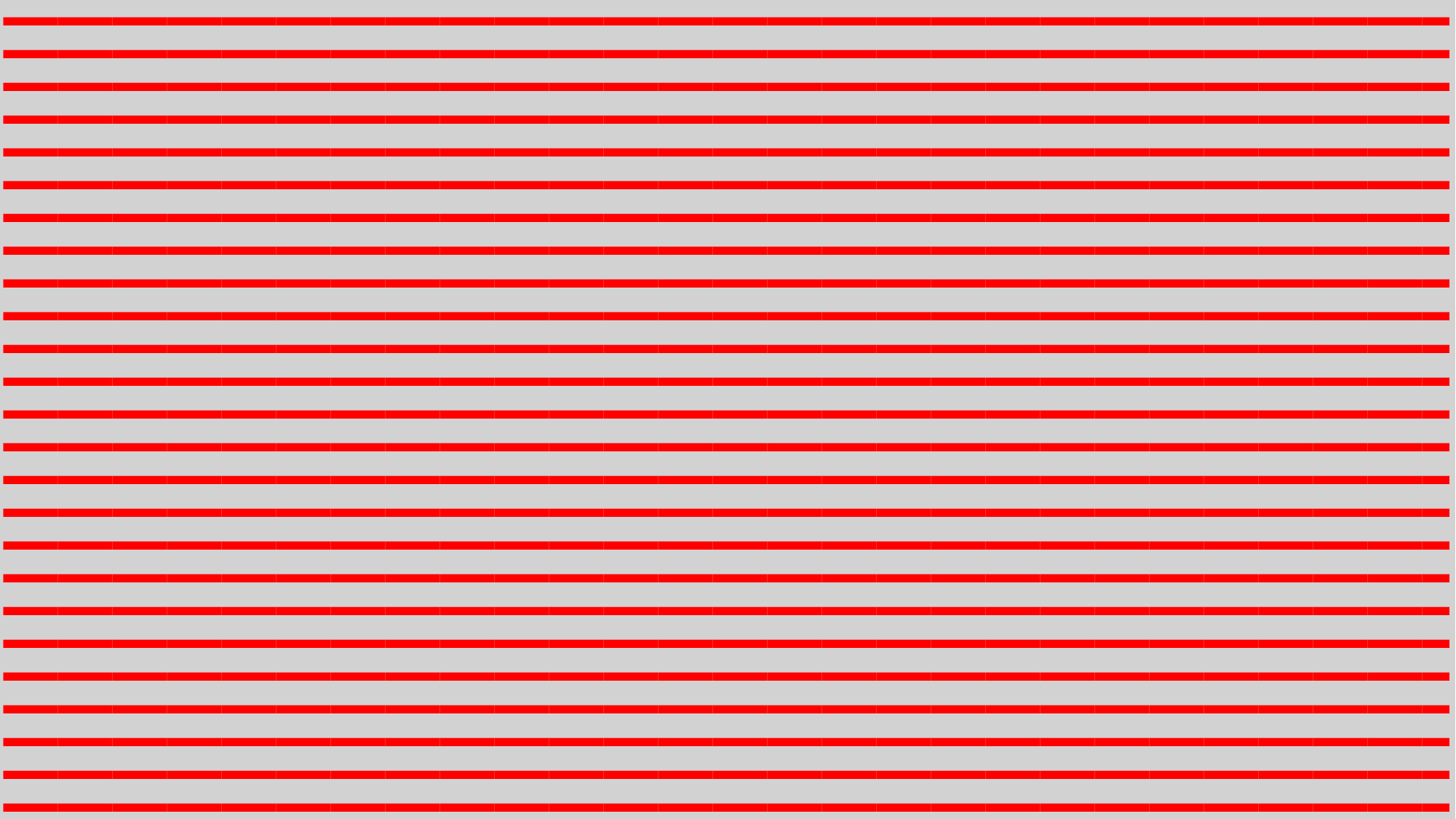

▬▬▬▬▬▬▬▬▬▬▬▬▬▬▬▬▬▬▬▬▬▬▬▬▬▬▬▬▬▬▬▬▬▬▬▬▬▬▬▬▬▬▬▬▬▬▬▬▬▬▬▬▬
▬▬▬▬▬▬▬▬▬▬▬▬▬▬▬▬▬▬▬▬▬▬▬▬▬▬▬▬▬▬▬▬▬▬▬▬▬▬▬▬▬▬▬▬▬▬▬▬▬▬▬▬▬
▬▬▬▬▬▬▬▬▬▬▬▬▬▬▬▬▬▬▬▬▬▬▬▬▬▬▬▬▬▬▬▬▬▬▬▬▬▬▬▬▬▬▬▬▬▬▬▬▬▬▬▬▬
▬▬▬▬▬▬▬▬▬▬▬▬▬▬▬▬▬▬▬▬▬▬▬▬▬▬▬▬▬▬▬▬▬▬▬▬▬▬▬▬▬▬▬▬▬▬▬▬▬▬▬▬▬
▬▬▬▬▬▬▬▬▬▬▬▬▬▬▬▬▬▬▬▬▬▬▬▬▬▬▬▬▬▬▬▬▬▬▬▬▬▬▬▬▬▬▬▬▬▬▬▬▬▬▬▬▬
▬▬▬▬▬▬▬▬▬▬▬▬▬▬▬▬▬▬▬▬▬▬▬▬▬▬▬▬▬▬▬▬▬▬▬▬▬▬▬▬▬▬▬▬▬▬▬▬▬▬▬▬▬
▬▬▬▬▬▬▬▬▬▬▬▬▬▬▬▬▬▬▬▬▬▬▬▬▬▬▬▬▬▬▬▬▬▬▬▬▬▬▬▬▬▬▬▬▬▬▬▬▬▬▬▬▬
▬▬▬▬▬▬▬▬▬▬▬▬▬▬▬▬▬▬▬▬▬▬▬▬▬▬▬▬▬▬▬▬▬▬▬▬▬▬▬▬▬▬▬▬▬▬▬▬▬▬▬▬▬
▬▬▬▬▬▬▬▬▬▬▬▬▬▬▬▬▬▬▬▬▬▬▬▬▬▬▬▬▬▬▬▬▬▬▬▬▬▬▬▬▬▬▬▬▬▬▬▬▬▬▬▬▬
▬▬▬▬▬▬▬▬▬▬▬▬▬▬▬▬▬▬▬▬▬▬▬▬▬▬▬▬▬▬▬▬▬▬▬▬▬▬▬▬▬▬▬▬▬▬▬▬▬▬▬▬▬
▬▬▬▬▬▬▬▬▬▬▬▬▬▬▬▬▬▬▬▬▬▬▬▬▬▬▬▬▬▬▬▬▬▬▬▬▬▬▬▬▬▬▬▬▬▬▬▬▬▬▬▬▬
▬▬▬▬▬▬▬▬▬▬▬▬▬▬▬▬▬▬▬▬▬▬▬▬▬▬▬▬▬▬▬▬▬▬▬▬▬▬▬▬▬▬▬▬▬▬▬▬▬▬▬▬▬
▬▬▬▬▬▬▬▬▬▬▬▬▬▬▬▬▬▬▬▬▬▬▬▬▬▬▬▬▬▬▬▬▬▬▬▬▬▬▬▬▬▬▬▬▬▬▬▬▬▬▬▬▬
▬▬▬▬▬▬▬▬▬▬▬▬▬▬▬▬▬▬▬▬▬▬▬▬▬▬▬▬▬▬▬▬▬▬▬▬▬▬▬▬▬▬▬▬▬▬▬▬▬▬▬▬▬
▬▬▬▬▬▬▬▬▬▬▬▬▬▬▬▬▬▬▬▬▬▬▬▬▬▬▬▬▬▬▬▬▬▬▬▬▬▬▬▬▬▬▬▬▬▬▬▬▬▬▬▬▬
▬▬▬▬▬▬▬▬▬▬▬▬▬▬▬▬▬▬▬▬▬▬▬▬▬▬▬▬▬▬▬▬▬▬▬▬▬▬▬▬▬▬▬▬▬▬▬▬▬▬▬▬▬
▬▬▬▬▬▬▬▬▬▬▬▬▬▬▬▬▬▬▬▬▬▬▬▬▬▬▬▬▬▬▬▬▬▬▬▬▬▬▬▬▬▬▬▬▬▬▬▬▬▬▬▬▬
▬▬▬▬▬▬▬▬▬▬▬▬▬▬▬▬▬▬▬▬▬▬▬▬▬▬▬▬▬▬▬▬▬▬▬▬▬▬▬▬▬▬▬▬▬▬▬▬▬▬▬▬▬
▬▬▬▬▬▬▬▬▬▬▬▬▬▬▬▬▬▬▬▬▬▬▬▬▬▬▬▬▬▬▬▬▬▬▬▬▬▬▬▬▬▬▬▬▬▬▬▬▬▬▬▬▬
▬▬▬▬▬▬▬▬▬▬▬▬▬▬▬▬▬▬▬▬▬▬▬▬▬▬▬▬▬▬▬▬▬▬▬▬▬▬▬▬▬▬▬▬▬▬▬▬▬▬▬▬▬
▬▬▬▬▬▬▬▬▬▬▬▬▬▬▬▬▬▬▬▬▬▬▬▬▬▬▬▬▬▬▬▬▬▬▬▬▬▬▬▬▬▬▬▬▬▬▬▬▬▬▬▬▬
▬▬▬▬▬▬▬▬▬▬▬▬▬▬▬▬▬▬▬▬▬▬▬▬▬▬▬▬▬▬▬▬▬▬▬▬▬▬▬▬▬▬▬▬▬▬▬▬▬▬▬▬▬
▬▬▬▬▬▬▬▬▬▬▬▬▬▬▬▬▬▬▬▬▬▬▬▬▬▬▬▬▬▬▬▬▬▬▬▬▬▬▬▬▬▬▬▬▬▬▬▬▬▬▬▬▬
▬▬▬▬▬▬▬▬▬▬▬▬▬▬▬▬▬▬▬▬▬▬▬▬▬▬▬▬▬▬▬▬▬▬▬▬▬▬▬▬▬▬▬▬▬▬▬▬▬▬▬▬▬
▬▬▬▬▬▬▬▬▬▬▬▬▬▬▬▬▬▬▬▬▬▬▬▬▬▬▬▬▬▬▬▬▬▬▬▬▬▬▬▬▬▬▬▬▬▬▬▬▬▬▬▬▬
▬▬▬▬▬▬▬▬▬▬▬▬▬▬▬▬▬▬▬▬▬▬▬▬▬▬▬▬▬▬▬▬▬▬▬▬▬▬▬▬▬▬▬▬▬▬▬▬▬▬▬▬▬
▬▬▬▬▬▬▬▬▬▬▬▬▬▬▬▬▬▬▬▬▬▬▬▬▬▬▬▬▬▬▬▬▬▬▬▬▬▬▬▬▬▬▬▬▬▬▬▬▬▬▬▬▬
▬▬▬▬▬▬▬▬▬▬▬▬▬▬▬▬▬▬▬▬▬▬▬▬▬▬▬▬▬▬▬▬▬▬▬▬▬▬▬▬▬▬▬▬▬▬▬▬▬▬▬▬▬
▬▬▬▬▬▬▬▬▬▬▬▬▬▬▬▬▬▬▬▬▬▬▬▬▬▬▬▬▬▬▬▬▬▬▬▬▬▬▬▬▬▬▬▬▬▬▬▬▬▬▬▬▬

## Slide 4
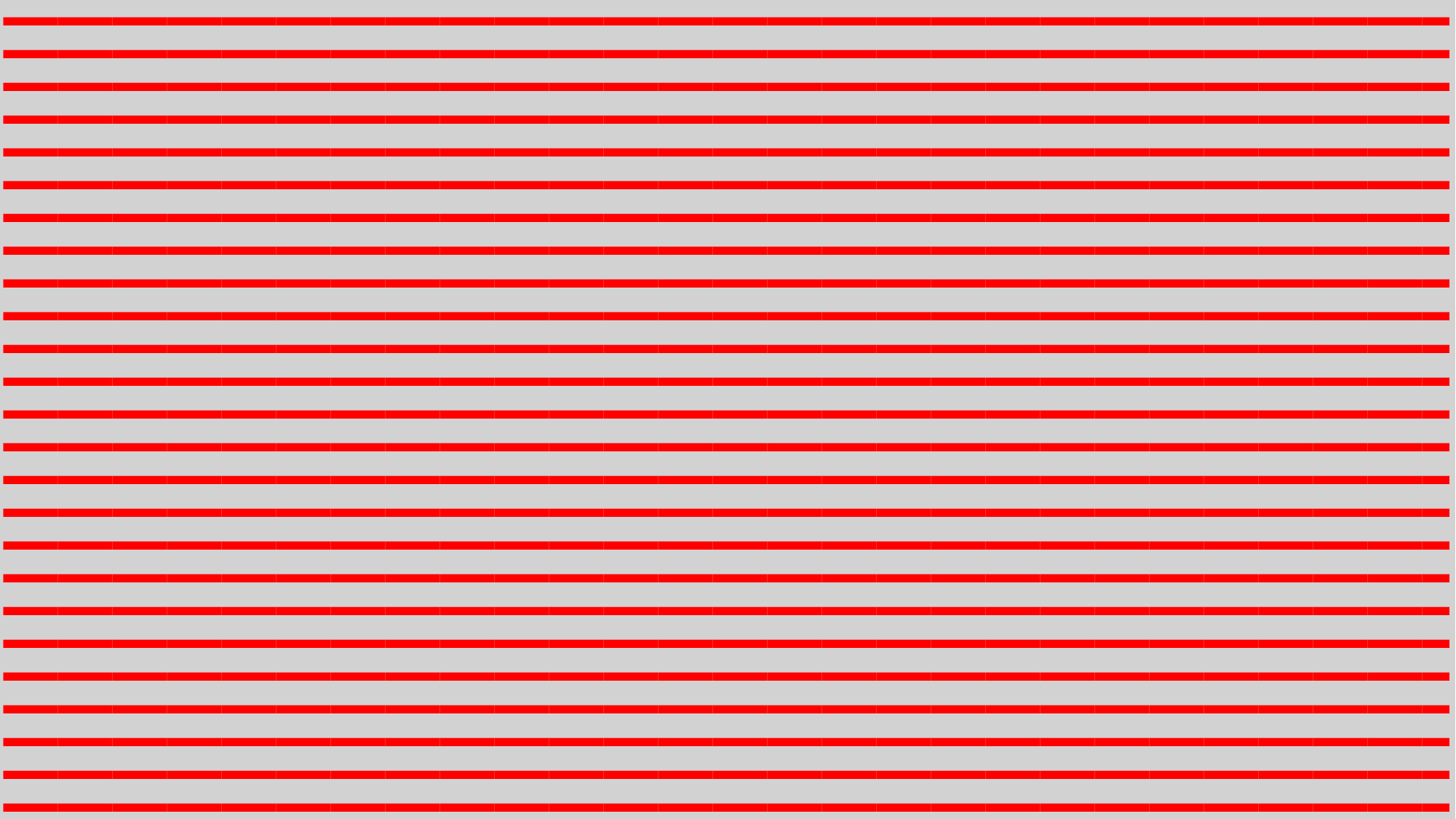

▬▬▬▬▬▬▬▬▬▬▬▬▬▬▬▬▬▬▬▬▬▬▬▬▬▬▬▬▬▬▬▬▬▬▬▬▬▬▬▬▬▬▬▬▬▬▬▬▬▬▬▬▬
▬▬▬▬▬▬▬▬▬▬▬▬▬▬▬▬▬▬▬▬▬▬▬▬▬▬▬▬▬▬▬▬▬▬▬▬▬▬▬▬▬▬▬▬▬▬▬▬▬▬▬▬▬
▬▬▬▬▬▬▬▬▬▬▬▬▬▬▬▬▬▬▬▬▬▬▬▬▬▬▬▬▬▬▬▬▬▬▬▬▬▬▬▬▬▬▬▬▬▬▬▬▬▬▬▬▬
▬▬▬▬▬▬▬▬▬▬▬▬▬▬▬▬▬▬▬▬▬▬▬▬▬▬▬▬▬▬▬▬▬▬▬▬▬▬▬▬▬▬▬▬▬▬▬▬▬▬▬▬▬
▬▬▬▬▬▬▬▬▬▬▬▬▬▬▬▬▬▬▬▬▬▬▬▬▬▬▬▬▬▬▬▬▬▬▬▬▬▬▬▬▬▬▬▬▬▬▬▬▬▬▬▬▬
▬▬▬▬▬▬▬▬▬▬▬▬▬▬▬▬▬▬▬▬▬▬▬▬▬▬▬▬▬▬▬▬▬▬▬▬▬▬▬▬▬▬▬▬▬▬▬▬▬▬▬▬▬
▬▬▬▬▬▬▬▬▬▬▬▬▬▬▬▬▬▬▬▬▬▬▬▬▬▬▬▬▬▬▬▬▬▬▬▬▬▬▬▬▬▬▬▬▬▬▬▬▬▬▬▬▬
▬▬▬▬▬▬▬▬▬▬▬▬▬▬▬▬▬▬▬▬▬▬▬▬▬▬▬▬▬▬▬▬▬▬▬▬▬▬▬▬▬▬▬▬▬▬▬▬▬▬▬▬▬
▬▬▬▬▬▬▬▬▬▬▬▬▬▬▬▬▬▬▬▬▬▬▬▬▬▬▬▬▬▬▬▬▬▬▬▬▬▬▬▬▬▬▬▬▬▬▬▬▬▬▬▬▬
▬▬▬▬▬▬▬▬▬▬▬▬▬▬▬▬▬▬▬▬▬▬▬▬▬▬▬▬▬▬▬▬▬▬▬▬▬▬▬▬▬▬▬▬▬▬▬▬▬▬▬▬▬
▬▬▬▬▬▬▬▬▬▬▬▬▬▬▬▬▬▬▬▬▬▬▬▬▬▬▬▬▬▬▬▬▬▬▬▬▬▬▬▬▬▬▬▬▬▬▬▬▬▬▬▬▬
▬▬▬▬▬▬▬▬▬▬▬▬▬▬▬▬▬▬▬▬▬▬▬▬▬▬▬▬▬▬▬▬▬▬▬▬▬▬▬▬▬▬▬▬▬▬▬▬▬▬▬▬▬
▬▬▬▬▬▬▬▬▬▬▬▬▬▬▬▬▬▬▬▬▬▬▬▬▬▬▬▬▬▬▬▬▬▬▬▬▬▬▬▬▬▬▬▬▬▬▬▬▬▬▬▬▬
▬▬▬▬▬▬▬▬▬▬▬▬▬▬▬▬▬▬▬▬▬▬▬▬▬▬▬▬▬▬▬▬▬▬▬▬▬▬▬▬▬▬▬▬▬▬▬▬▬▬▬▬▬
▬▬▬▬▬▬▬▬▬▬▬▬▬▬▬▬▬▬▬▬▬▬▬▬▬▬▬▬▬▬▬▬▬▬▬▬▬▬▬▬▬▬▬▬▬▬▬▬▬▬▬▬▬
▬▬▬▬▬▬▬▬▬▬▬▬▬▬▬▬▬▬▬▬▬▬▬▬▬▬▬▬▬▬▬▬▬▬▬▬▬▬▬▬▬▬▬▬▬▬▬▬▬▬▬▬▬
▬▬▬▬▬▬▬▬▬▬▬▬▬▬▬▬▬▬▬▬▬▬▬▬▬▬▬▬▬▬▬▬▬▬▬▬▬▬▬▬▬▬▬▬▬▬▬▬▬▬▬▬▬
▬▬▬▬▬▬▬▬▬▬▬▬▬▬▬▬▬▬▬▬▬▬▬▬▬▬▬▬▬▬▬▬▬▬▬▬▬▬▬▬▬▬▬▬▬▬▬▬▬▬▬▬▬
▬▬▬▬▬▬▬▬▬▬▬▬▬▬▬▬▬▬▬▬▬▬▬▬▬▬▬▬▬▬▬▬▬▬▬▬▬▬▬▬▬▬▬▬▬▬▬▬▬▬▬▬▬
▬▬▬▬▬▬▬▬▬▬▬▬▬▬▬▬▬▬▬▬▬▬▬▬▬▬▬▬▬▬▬▬▬▬▬▬▬▬▬▬▬▬▬▬▬▬▬▬▬▬▬▬▬
▬▬▬▬▬▬▬▬▬▬▬▬▬▬▬▬▬▬▬▬▬▬▬▬▬▬▬▬▬▬▬▬▬▬▬▬▬▬▬▬▬▬▬▬▬▬▬▬▬▬▬▬▬
▬▬▬▬▬▬▬▬▬▬▬▬▬▬▬▬▬▬▬▬▬▬▬▬▬▬▬▬▬▬▬▬▬▬▬▬▬▬▬▬▬▬▬▬▬▬▬▬▬▬▬▬▬
▬▬▬▬▬▬▬▬▬▬▬▬▬▬▬▬▬▬▬▬▬▬▬▬▬▬▬▬▬▬▬▬▬▬▬▬▬▬▬▬▬▬▬▬▬▬▬▬▬▬▬▬▬
▬▬▬▬▬▬▬▬▬▬▬▬▬▬▬▬▬▬▬▬▬▬▬▬▬▬▬▬▬▬▬▬▬▬▬▬▬▬▬▬▬▬▬▬▬▬▬▬▬▬▬▬▬
▬▬▬▬▬▬▬▬▬▬▬▬▬▬▬▬▬▬▬▬▬▬▬▬▬▬▬▬▬▬▬▬▬▬▬▬▬▬▬▬▬▬▬▬▬▬▬▬▬▬▬▬▬
▬▬▬▬▬▬▬▬▬▬▬▬▬▬▬▬▬▬▬▬▬▬▬▬▬▬▬▬▬▬▬▬▬▬▬▬▬▬▬▬▬▬▬▬▬▬▬▬▬▬▬▬▬
▬▬▬▬▬▬▬▬▬▬▬▬▬▬▬▬▬▬▬▬▬▬▬▬▬▬▬▬▬▬▬▬▬▬▬▬▬▬▬▬▬▬▬▬▬▬▬▬▬▬▬▬▬
▬▬▬▬▬▬▬▬▬▬▬▬▬▬▬▬▬▬▬▬▬▬▬▬▬▬▬▬▬▬▬▬▬▬▬▬▬▬▬▬▬▬▬▬▬▬▬▬▬▬▬▬▬
▬▬▬▬▬▬▬▬▬▬▬▬▬▬▬▬▬▬▬▬▬▬▬▬▬▬▬▬▬▬▬▬▬▬▬▬▬▬▬▬▬▬▬▬▬▬▬▬▬▬▬▬▬

## Slide 5
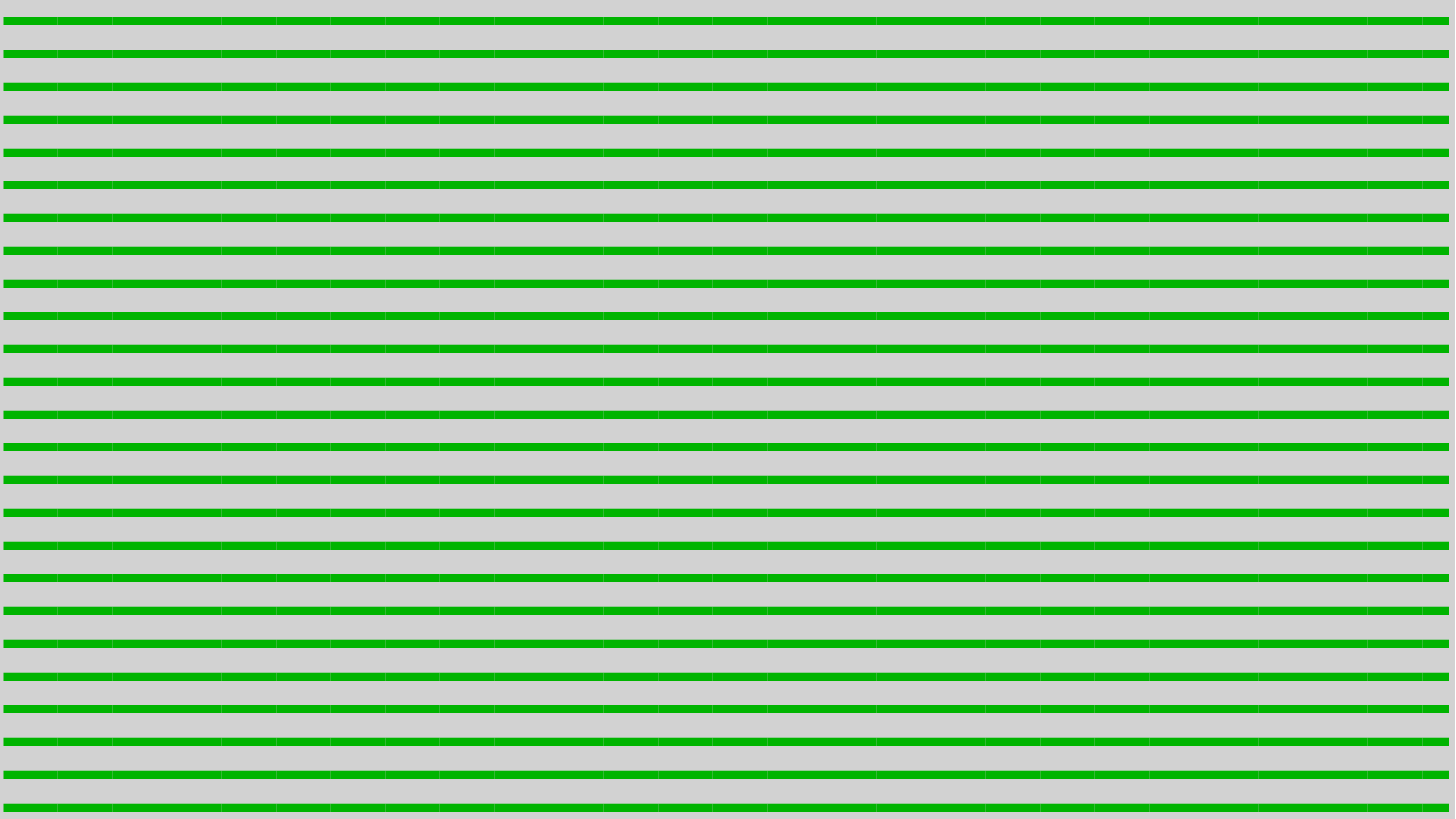

▬▬▬▬▬▬▬▬▬▬▬▬▬▬▬▬▬▬▬▬▬▬▬▬▬▬▬▬▬▬▬▬▬▬▬▬▬▬▬▬▬▬▬▬▬▬▬▬▬▬▬▬▬
▬▬▬▬▬▬▬▬▬▬▬▬▬▬▬▬▬▬▬▬▬▬▬▬▬▬▬▬▬▬▬▬▬▬▬▬▬▬▬▬▬▬▬▬▬▬▬▬▬▬▬▬▬
▬▬▬▬▬▬▬▬▬▬▬▬▬▬▬▬▬▬▬▬▬▬▬▬▬▬▬▬▬▬▬▬▬▬▬▬▬▬▬▬▬▬▬▬▬▬▬▬▬▬▬▬▬
▬▬▬▬▬▬▬▬▬▬▬▬▬▬▬▬▬▬▬▬▬▬▬▬▬▬▬▬▬▬▬▬▬▬▬▬▬▬▬▬▬▬▬▬▬▬▬▬▬▬▬▬▬
▬▬▬▬▬▬▬▬▬▬▬▬▬▬▬▬▬▬▬▬▬▬▬▬▬▬▬▬▬▬▬▬▬▬▬▬▬▬▬▬▬▬▬▬▬▬▬▬▬▬▬▬▬
▬▬▬▬▬▬▬▬▬▬▬▬▬▬▬▬▬▬▬▬▬▬▬▬▬▬▬▬▬▬▬▬▬▬▬▬▬▬▬▬▬▬▬▬▬▬▬▬▬▬▬▬▬
▬▬▬▬▬▬▬▬▬▬▬▬▬▬▬▬▬▬▬▬▬▬▬▬▬▬▬▬▬▬▬▬▬▬▬▬▬▬▬▬▬▬▬▬▬▬▬▬▬▬▬▬▬
▬▬▬▬▬▬▬▬▬▬▬▬▬▬▬▬▬▬▬▬▬▬▬▬▬▬▬▬▬▬▬▬▬▬▬▬▬▬▬▬▬▬▬▬▬▬▬▬▬▬▬▬▬
▬▬▬▬▬▬▬▬▬▬▬▬▬▬▬▬▬▬▬▬▬▬▬▬▬▬▬▬▬▬▬▬▬▬▬▬▬▬▬▬▬▬▬▬▬▬▬▬▬▬▬▬▬
▬▬▬▬▬▬▬▬▬▬▬▬▬▬▬▬▬▬▬▬▬▬▬▬▬▬▬▬▬▬▬▬▬▬▬▬▬▬▬▬▬▬▬▬▬▬▬▬▬▬▬▬▬
▬▬▬▬▬▬▬▬▬▬▬▬▬▬▬▬▬▬▬▬▬▬▬▬▬▬▬▬▬▬▬▬▬▬▬▬▬▬▬▬▬▬▬▬▬▬▬▬▬▬▬▬▬
▬▬▬▬▬▬▬▬▬▬▬▬▬▬▬▬▬▬▬▬▬▬▬▬▬▬▬▬▬▬▬▬▬▬▬▬▬▬▬▬▬▬▬▬▬▬▬▬▬▬▬▬▬
▬▬▬▬▬▬▬▬▬▬▬▬▬▬▬▬▬▬▬▬▬▬▬▬▬▬▬▬▬▬▬▬▬▬▬▬▬▬▬▬▬▬▬▬▬▬▬▬▬▬▬▬▬
▬▬▬▬▬▬▬▬▬▬▬▬▬▬▬▬▬▬▬▬▬▬▬▬▬▬▬▬▬▬▬▬▬▬▬▬▬▬▬▬▬▬▬▬▬▬▬▬▬▬▬▬▬
▬▬▬▬▬▬▬▬▬▬▬▬▬▬▬▬▬▬▬▬▬▬▬▬▬▬▬▬▬▬▬▬▬▬▬▬▬▬▬▬▬▬▬▬▬▬▬▬▬▬▬▬▬
▬▬▬▬▬▬▬▬▬▬▬▬▬▬▬▬▬▬▬▬▬▬▬▬▬▬▬▬▬▬▬▬▬▬▬▬▬▬▬▬▬▬▬▬▬▬▬▬▬▬▬▬▬
▬▬▬▬▬▬▬▬▬▬▬▬▬▬▬▬▬▬▬▬▬▬▬▬▬▬▬▬▬▬▬▬▬▬▬▬▬▬▬▬▬▬▬▬▬▬▬▬▬▬▬▬▬
▬▬▬▬▬▬▬▬▬▬▬▬▬▬▬▬▬▬▬▬▬▬▬▬▬▬▬▬▬▬▬▬▬▬▬▬▬▬▬▬▬▬▬▬▬▬▬▬▬▬▬▬▬
▬▬▬▬▬▬▬▬▬▬▬▬▬▬▬▬▬▬▬▬▬▬▬▬▬▬▬▬▬▬▬▬▬▬▬▬▬▬▬▬▬▬▬▬▬▬▬▬▬▬▬▬▬
▬▬▬▬▬▬▬▬▬▬▬▬▬▬▬▬▬▬▬▬▬▬▬▬▬▬▬▬▬▬▬▬▬▬▬▬▬▬▬▬▬▬▬▬▬▬▬▬▬▬▬▬▬
▬▬▬▬▬▬▬▬▬▬▬▬▬▬▬▬▬▬▬▬▬▬▬▬▬▬▬▬▬▬▬▬▬▬▬▬▬▬▬▬▬▬▬▬▬▬▬▬▬▬▬▬▬
▬▬▬▬▬▬▬▬▬▬▬▬▬▬▬▬▬▬▬▬▬▬▬▬▬▬▬▬▬▬▬▬▬▬▬▬▬▬▬▬▬▬▬▬▬▬▬▬▬▬▬▬▬
▬▬▬▬▬▬▬▬▬▬▬▬▬▬▬▬▬▬▬▬▬▬▬▬▬▬▬▬▬▬▬▬▬▬▬▬▬▬▬▬▬▬▬▬▬▬▬▬▬▬▬▬▬
▬▬▬▬▬▬▬▬▬▬▬▬▬▬▬▬▬▬▬▬▬▬▬▬▬▬▬▬▬▬▬▬▬▬▬▬▬▬▬▬▬▬▬▬▬▬▬▬▬▬▬▬▬
▬▬▬▬▬▬▬▬▬▬▬▬▬▬▬▬▬▬▬▬▬▬▬▬▬▬▬▬▬▬▬▬▬▬▬▬▬▬▬▬▬▬▬▬▬▬▬▬▬▬▬▬▬
▬▬▬▬▬▬▬▬▬▬▬▬▬▬▬▬▬▬▬▬▬▬▬▬▬▬▬▬▬▬▬▬▬▬▬▬▬▬▬▬▬▬▬▬▬▬▬▬▬▬▬▬▬
▬▬▬▬▬▬▬▬▬▬▬▬▬▬▬▬▬▬▬▬▬▬▬▬▬▬▬▬▬▬▬▬▬▬▬▬▬▬▬▬▬▬▬▬▬▬▬▬▬▬▬▬▬
▬▬▬▬▬▬▬▬▬▬▬▬▬▬▬▬▬▬▬▬▬▬▬▬▬▬▬▬▬▬▬▬▬▬▬▬▬▬▬▬▬▬▬▬▬▬▬▬▬▬▬▬▬
▬▬▬▬▬▬▬▬▬▬▬▬▬▬▬▬▬▬▬▬▬▬▬▬▬▬▬▬▬▬▬▬▬▬▬▬▬▬▬▬▬▬▬▬▬▬▬▬▬▬▬▬▬

## Slide 6
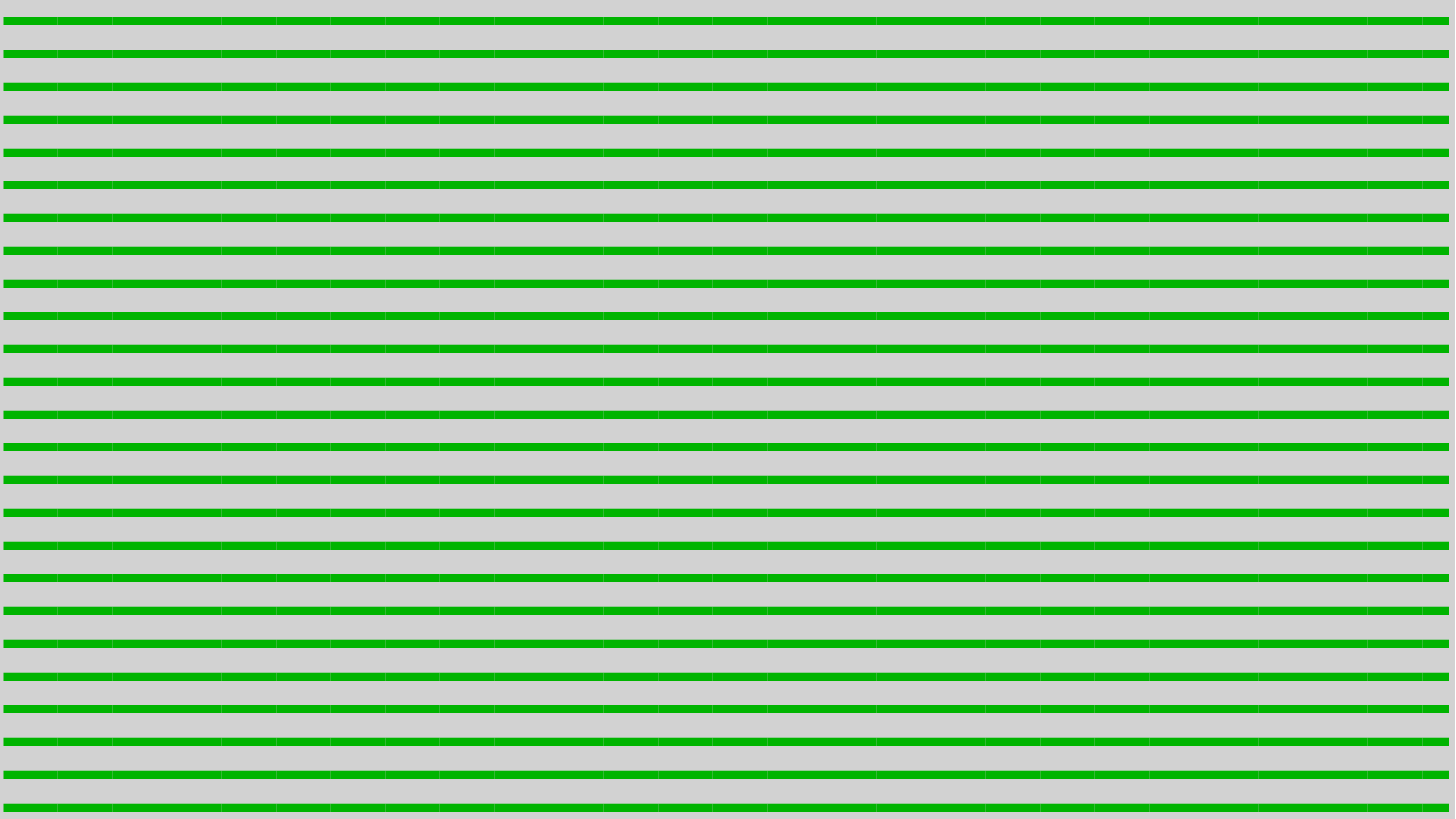

▬▬▬▬▬▬▬▬▬▬▬▬▬▬▬▬▬▬▬▬▬▬▬▬▬▬▬▬▬▬▬▬▬▬▬▬▬▬▬▬▬▬▬▬▬▬▬▬▬▬▬▬▬
▬▬▬▬▬▬▬▬▬▬▬▬▬▬▬▬▬▬▬▬▬▬▬▬▬▬▬▬▬▬▬▬▬▬▬▬▬▬▬▬▬▬▬▬▬▬▬▬▬▬▬▬▬
▬▬▬▬▬▬▬▬▬▬▬▬▬▬▬▬▬▬▬▬▬▬▬▬▬▬▬▬▬▬▬▬▬▬▬▬▬▬▬▬▬▬▬▬▬▬▬▬▬▬▬▬▬
▬▬▬▬▬▬▬▬▬▬▬▬▬▬▬▬▬▬▬▬▬▬▬▬▬▬▬▬▬▬▬▬▬▬▬▬▬▬▬▬▬▬▬▬▬▬▬▬▬▬▬▬▬
▬▬▬▬▬▬▬▬▬▬▬▬▬▬▬▬▬▬▬▬▬▬▬▬▬▬▬▬▬▬▬▬▬▬▬▬▬▬▬▬▬▬▬▬▬▬▬▬▬▬▬▬▬
▬▬▬▬▬▬▬▬▬▬▬▬▬▬▬▬▬▬▬▬▬▬▬▬▬▬▬▬▬▬▬▬▬▬▬▬▬▬▬▬▬▬▬▬▬▬▬▬▬▬▬▬▬
▬▬▬▬▬▬▬▬▬▬▬▬▬▬▬▬▬▬▬▬▬▬▬▬▬▬▬▬▬▬▬▬▬▬▬▬▬▬▬▬▬▬▬▬▬▬▬▬▬▬▬▬▬
▬▬▬▬▬▬▬▬▬▬▬▬▬▬▬▬▬▬▬▬▬▬▬▬▬▬▬▬▬▬▬▬▬▬▬▬▬▬▬▬▬▬▬▬▬▬▬▬▬▬▬▬▬
▬▬▬▬▬▬▬▬▬▬▬▬▬▬▬▬▬▬▬▬▬▬▬▬▬▬▬▬▬▬▬▬▬▬▬▬▬▬▬▬▬▬▬▬▬▬▬▬▬▬▬▬▬
▬▬▬▬▬▬▬▬▬▬▬▬▬▬▬▬▬▬▬▬▬▬▬▬▬▬▬▬▬▬▬▬▬▬▬▬▬▬▬▬▬▬▬▬▬▬▬▬▬▬▬▬▬
▬▬▬▬▬▬▬▬▬▬▬▬▬▬▬▬▬▬▬▬▬▬▬▬▬▬▬▬▬▬▬▬▬▬▬▬▬▬▬▬▬▬▬▬▬▬▬▬▬▬▬▬▬
▬▬▬▬▬▬▬▬▬▬▬▬▬▬▬▬▬▬▬▬▬▬▬▬▬▬▬▬▬▬▬▬▬▬▬▬▬▬▬▬▬▬▬▬▬▬▬▬▬▬▬▬▬
▬▬▬▬▬▬▬▬▬▬▬▬▬▬▬▬▬▬▬▬▬▬▬▬▬▬▬▬▬▬▬▬▬▬▬▬▬▬▬▬▬▬▬▬▬▬▬▬▬▬▬▬▬
▬▬▬▬▬▬▬▬▬▬▬▬▬▬▬▬▬▬▬▬▬▬▬▬▬▬▬▬▬▬▬▬▬▬▬▬▬▬▬▬▬▬▬▬▬▬▬▬▬▬▬▬▬
▬▬▬▬▬▬▬▬▬▬▬▬▬▬▬▬▬▬▬▬▬▬▬▬▬▬▬▬▬▬▬▬▬▬▬▬▬▬▬▬▬▬▬▬▬▬▬▬▬▬▬▬▬
▬▬▬▬▬▬▬▬▬▬▬▬▬▬▬▬▬▬▬▬▬▬▬▬▬▬▬▬▬▬▬▬▬▬▬▬▬▬▬▬▬▬▬▬▬▬▬▬▬▬▬▬▬
▬▬▬▬▬▬▬▬▬▬▬▬▬▬▬▬▬▬▬▬▬▬▬▬▬▬▬▬▬▬▬▬▬▬▬▬▬▬▬▬▬▬▬▬▬▬▬▬▬▬▬▬▬
▬▬▬▬▬▬▬▬▬▬▬▬▬▬▬▬▬▬▬▬▬▬▬▬▬▬▬▬▬▬▬▬▬▬▬▬▬▬▬▬▬▬▬▬▬▬▬▬▬▬▬▬▬
▬▬▬▬▬▬▬▬▬▬▬▬▬▬▬▬▬▬▬▬▬▬▬▬▬▬▬▬▬▬▬▬▬▬▬▬▬▬▬▬▬▬▬▬▬▬▬▬▬▬▬▬▬
▬▬▬▬▬▬▬▬▬▬▬▬▬▬▬▬▬▬▬▬▬▬▬▬▬▬▬▬▬▬▬▬▬▬▬▬▬▬▬▬▬▬▬▬▬▬▬▬▬▬▬▬▬
▬▬▬▬▬▬▬▬▬▬▬▬▬▬▬▬▬▬▬▬▬▬▬▬▬▬▬▬▬▬▬▬▬▬▬▬▬▬▬▬▬▬▬▬▬▬▬▬▬▬▬▬▬
▬▬▬▬▬▬▬▬▬▬▬▬▬▬▬▬▬▬▬▬▬▬▬▬▬▬▬▬▬▬▬▬▬▬▬▬▬▬▬▬▬▬▬▬▬▬▬▬▬▬▬▬▬
▬▬▬▬▬▬▬▬▬▬▬▬▬▬▬▬▬▬▬▬▬▬▬▬▬▬▬▬▬▬▬▬▬▬▬▬▬▬▬▬▬▬▬▬▬▬▬▬▬▬▬▬▬
▬▬▬▬▬▬▬▬▬▬▬▬▬▬▬▬▬▬▬▬▬▬▬▬▬▬▬▬▬▬▬▬▬▬▬▬▬▬▬▬▬▬▬▬▬▬▬▬▬▬▬▬▬
▬▬▬▬▬▬▬▬▬▬▬▬▬▬▬▬▬▬▬▬▬▬▬▬▬▬▬▬▬▬▬▬▬▬▬▬▬▬▬▬▬▬▬▬▬▬▬▬▬▬▬▬▬
▬▬▬▬▬▬▬▬▬▬▬▬▬▬▬▬▬▬▬▬▬▬▬▬▬▬▬▬▬▬▬▬▬▬▬▬▬▬▬▬▬▬▬▬▬▬▬▬▬▬▬▬▬
▬▬▬▬▬▬▬▬▬▬▬▬▬▬▬▬▬▬▬▬▬▬▬▬▬▬▬▬▬▬▬▬▬▬▬▬▬▬▬▬▬▬▬▬▬▬▬▬▬▬▬▬▬
▬▬▬▬▬▬▬▬▬▬▬▬▬▬▬▬▬▬▬▬▬▬▬▬▬▬▬▬▬▬▬▬▬▬▬▬▬▬▬▬▬▬▬▬▬▬▬▬▬▬▬▬▬
▬▬▬▬▬▬▬▬▬▬▬▬▬▬▬▬▬▬▬▬▬▬▬▬▬▬▬▬▬▬▬▬▬▬▬▬▬▬▬▬▬▬▬▬▬▬▬▬▬▬▬▬▬

## Slide 7
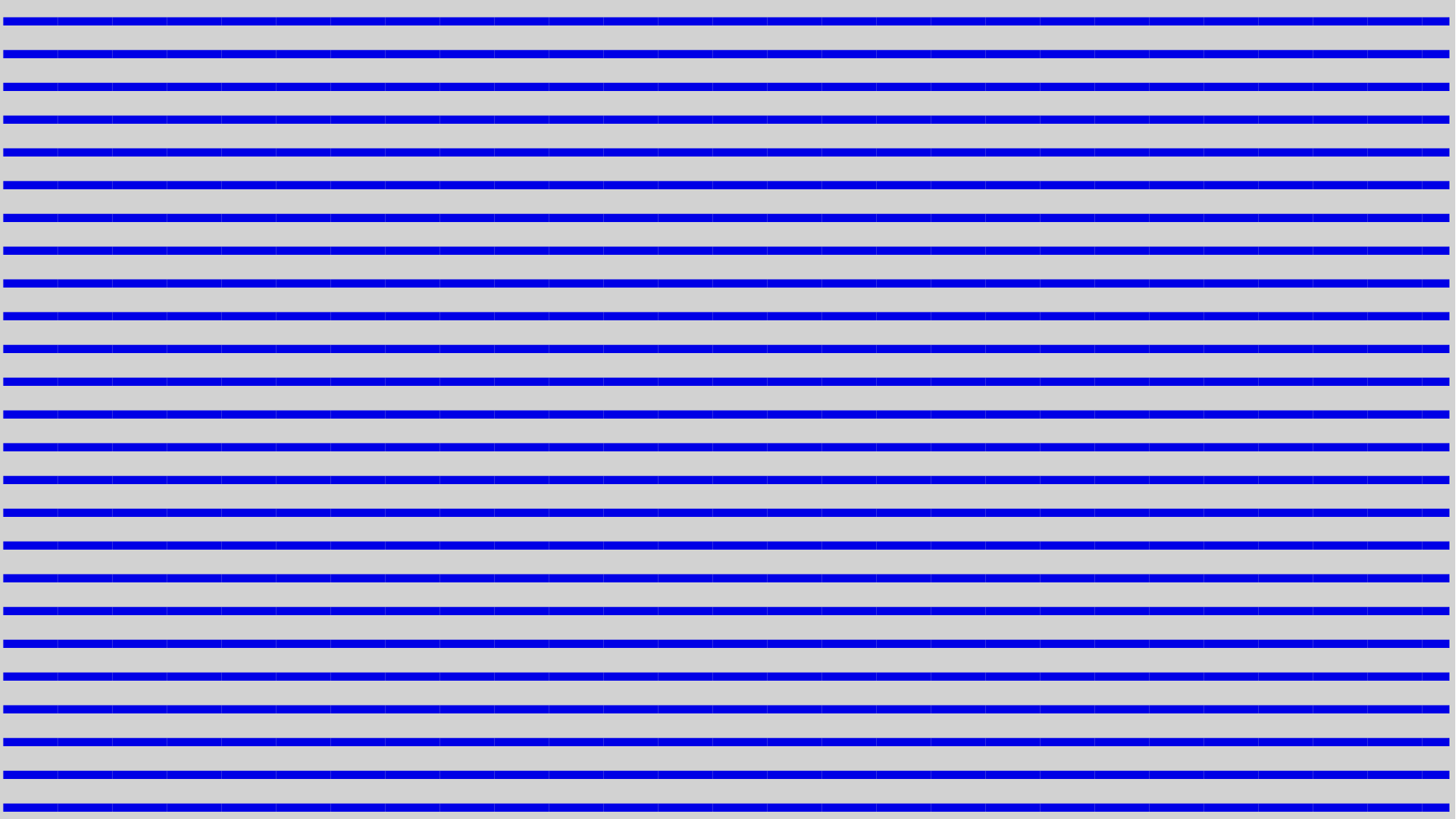

▬▬▬▬▬▬▬▬▬▬▬▬▬▬▬▬▬▬▬▬▬▬▬▬▬▬▬▬▬▬▬▬▬▬▬▬▬▬▬▬▬▬▬▬▬▬▬▬▬▬▬▬▬
▬▬▬▬▬▬▬▬▬▬▬▬▬▬▬▬▬▬▬▬▬▬▬▬▬▬▬▬▬▬▬▬▬▬▬▬▬▬▬▬▬▬▬▬▬▬▬▬▬▬▬▬▬
▬▬▬▬▬▬▬▬▬▬▬▬▬▬▬▬▬▬▬▬▬▬▬▬▬▬▬▬▬▬▬▬▬▬▬▬▬▬▬▬▬▬▬▬▬▬▬▬▬▬▬▬▬
▬▬▬▬▬▬▬▬▬▬▬▬▬▬▬▬▬▬▬▬▬▬▬▬▬▬▬▬▬▬▬▬▬▬▬▬▬▬▬▬▬▬▬▬▬▬▬▬▬▬▬▬▬
▬▬▬▬▬▬▬▬▬▬▬▬▬▬▬▬▬▬▬▬▬▬▬▬▬▬▬▬▬▬▬▬▬▬▬▬▬▬▬▬▬▬▬▬▬▬▬▬▬▬▬▬▬
▬▬▬▬▬▬▬▬▬▬▬▬▬▬▬▬▬▬▬▬▬▬▬▬▬▬▬▬▬▬▬▬▬▬▬▬▬▬▬▬▬▬▬▬▬▬▬▬▬▬▬▬▬
▬▬▬▬▬▬▬▬▬▬▬▬▬▬▬▬▬▬▬▬▬▬▬▬▬▬▬▬▬▬▬▬▬▬▬▬▬▬▬▬▬▬▬▬▬▬▬▬▬▬▬▬▬
▬▬▬▬▬▬▬▬▬▬▬▬▬▬▬▬▬▬▬▬▬▬▬▬▬▬▬▬▬▬▬▬▬▬▬▬▬▬▬▬▬▬▬▬▬▬▬▬▬▬▬▬▬
▬▬▬▬▬▬▬▬▬▬▬▬▬▬▬▬▬▬▬▬▬▬▬▬▬▬▬▬▬▬▬▬▬▬▬▬▬▬▬▬▬▬▬▬▬▬▬▬▬▬▬▬▬
▬▬▬▬▬▬▬▬▬▬▬▬▬▬▬▬▬▬▬▬▬▬▬▬▬▬▬▬▬▬▬▬▬▬▬▬▬▬▬▬▬▬▬▬▬▬▬▬▬▬▬▬▬
▬▬▬▬▬▬▬▬▬▬▬▬▬▬▬▬▬▬▬▬▬▬▬▬▬▬▬▬▬▬▬▬▬▬▬▬▬▬▬▬▬▬▬▬▬▬▬▬▬▬▬▬▬
▬▬▬▬▬▬▬▬▬▬▬▬▬▬▬▬▬▬▬▬▬▬▬▬▬▬▬▬▬▬▬▬▬▬▬▬▬▬▬▬▬▬▬▬▬▬▬▬▬▬▬▬▬
▬▬▬▬▬▬▬▬▬▬▬▬▬▬▬▬▬▬▬▬▬▬▬▬▬▬▬▬▬▬▬▬▬▬▬▬▬▬▬▬▬▬▬▬▬▬▬▬▬▬▬▬▬
▬▬▬▬▬▬▬▬▬▬▬▬▬▬▬▬▬▬▬▬▬▬▬▬▬▬▬▬▬▬▬▬▬▬▬▬▬▬▬▬▬▬▬▬▬▬▬▬▬▬▬▬▬
▬▬▬▬▬▬▬▬▬▬▬▬▬▬▬▬▬▬▬▬▬▬▬▬▬▬▬▬▬▬▬▬▬▬▬▬▬▬▬▬▬▬▬▬▬▬▬▬▬▬▬▬▬
▬▬▬▬▬▬▬▬▬▬▬▬▬▬▬▬▬▬▬▬▬▬▬▬▬▬▬▬▬▬▬▬▬▬▬▬▬▬▬▬▬▬▬▬▬▬▬▬▬▬▬▬▬
▬▬▬▬▬▬▬▬▬▬▬▬▬▬▬▬▬▬▬▬▬▬▬▬▬▬▬▬▬▬▬▬▬▬▬▬▬▬▬▬▬▬▬▬▬▬▬▬▬▬▬▬▬
▬▬▬▬▬▬▬▬▬▬▬▬▬▬▬▬▬▬▬▬▬▬▬▬▬▬▬▬▬▬▬▬▬▬▬▬▬▬▬▬▬▬▬▬▬▬▬▬▬▬▬▬▬
▬▬▬▬▬▬▬▬▬▬▬▬▬▬▬▬▬▬▬▬▬▬▬▬▬▬▬▬▬▬▬▬▬▬▬▬▬▬▬▬▬▬▬▬▬▬▬▬▬▬▬▬▬
▬▬▬▬▬▬▬▬▬▬▬▬▬▬▬▬▬▬▬▬▬▬▬▬▬▬▬▬▬▬▬▬▬▬▬▬▬▬▬▬▬▬▬▬▬▬▬▬▬▬▬▬▬
▬▬▬▬▬▬▬▬▬▬▬▬▬▬▬▬▬▬▬▬▬▬▬▬▬▬▬▬▬▬▬▬▬▬▬▬▬▬▬▬▬▬▬▬▬▬▬▬▬▬▬▬▬
▬▬▬▬▬▬▬▬▬▬▬▬▬▬▬▬▬▬▬▬▬▬▬▬▬▬▬▬▬▬▬▬▬▬▬▬▬▬▬▬▬▬▬▬▬▬▬▬▬▬▬▬▬
▬▬▬▬▬▬▬▬▬▬▬▬▬▬▬▬▬▬▬▬▬▬▬▬▬▬▬▬▬▬▬▬▬▬▬▬▬▬▬▬▬▬▬▬▬▬▬▬▬▬▬▬▬
▬▬▬▬▬▬▬▬▬▬▬▬▬▬▬▬▬▬▬▬▬▬▬▬▬▬▬▬▬▬▬▬▬▬▬▬▬▬▬▬▬▬▬▬▬▬▬▬▬▬▬▬▬
▬▬▬▬▬▬▬▬▬▬▬▬▬▬▬▬▬▬▬▬▬▬▬▬▬▬▬▬▬▬▬▬▬▬▬▬▬▬▬▬▬▬▬▬▬▬▬▬▬▬▬▬▬
▬▬▬▬▬▬▬▬▬▬▬▬▬▬▬▬▬▬▬▬▬▬▬▬▬▬▬▬▬▬▬▬▬▬▬▬▬▬▬▬▬▬▬▬▬▬▬▬▬▬▬▬▬
▬▬▬▬▬▬▬▬▬▬▬▬▬▬▬▬▬▬▬▬▬▬▬▬▬▬▬▬▬▬▬▬▬▬▬▬▬▬▬▬▬▬▬▬▬▬▬▬▬▬▬▬▬
▬▬▬▬▬▬▬▬▬▬▬▬▬▬▬▬▬▬▬▬▬▬▬▬▬▬▬▬▬▬▬▬▬▬▬▬▬▬▬▬▬▬▬▬▬▬▬▬▬▬▬▬▬
▬▬▬▬▬▬▬▬▬▬▬▬▬▬▬▬▬▬▬▬▬▬▬▬▬▬▬▬▬▬▬▬▬▬▬▬▬▬▬▬▬▬▬▬▬▬▬▬▬▬▬▬▬

## Slide 8
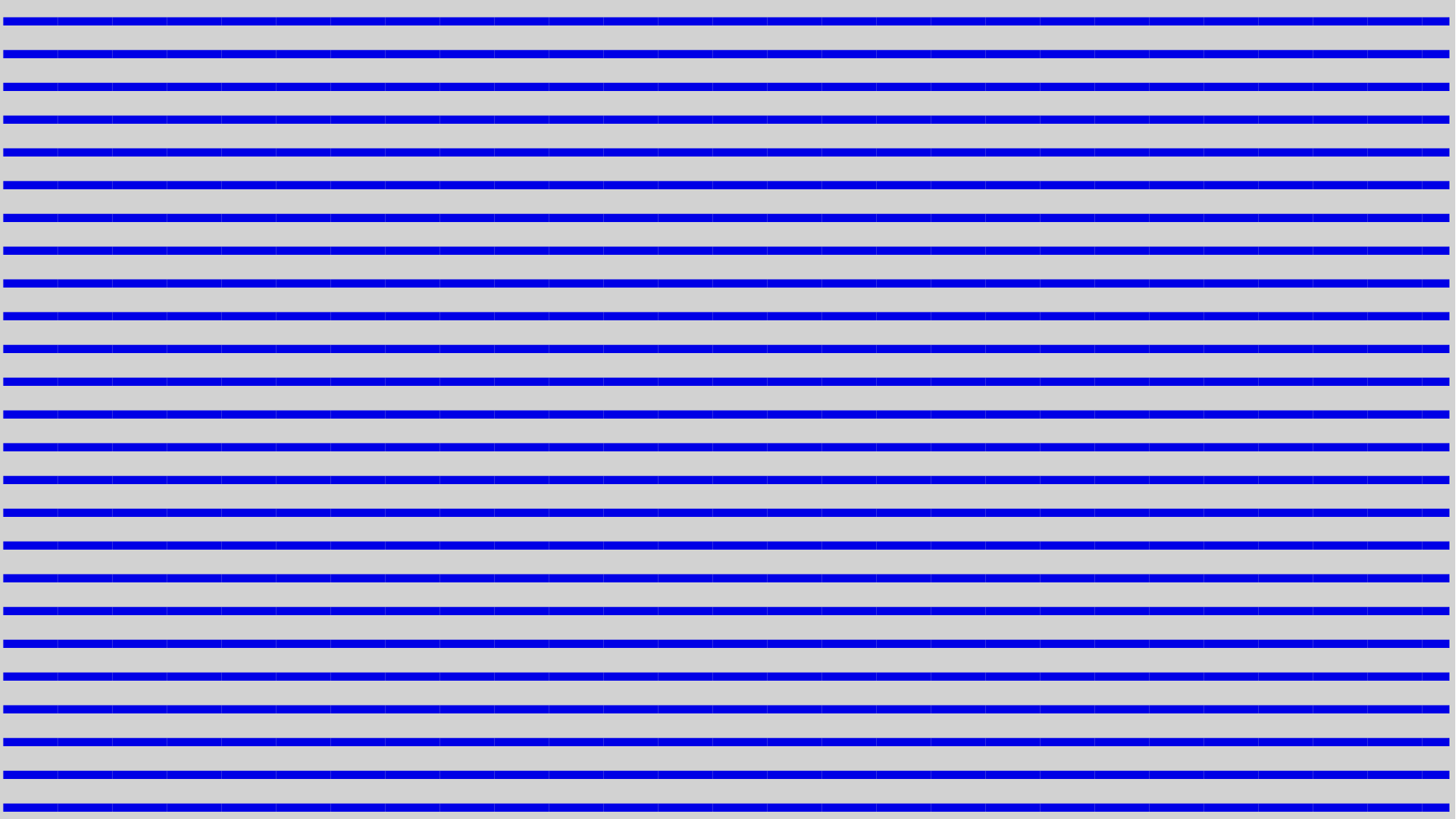

▬▬▬▬▬▬▬▬▬▬▬▬▬▬▬▬▬▬▬▬▬▬▬▬▬▬▬▬▬▬▬▬▬▬▬▬▬▬▬▬▬▬▬▬▬▬▬▬▬▬▬▬▬
▬▬▬▬▬▬▬▬▬▬▬▬▬▬▬▬▬▬▬▬▬▬▬▬▬▬▬▬▬▬▬▬▬▬▬▬▬▬▬▬▬▬▬▬▬▬▬▬▬▬▬▬▬
▬▬▬▬▬▬▬▬▬▬▬▬▬▬▬▬▬▬▬▬▬▬▬▬▬▬▬▬▬▬▬▬▬▬▬▬▬▬▬▬▬▬▬▬▬▬▬▬▬▬▬▬▬
▬▬▬▬▬▬▬▬▬▬▬▬▬▬▬▬▬▬▬▬▬▬▬▬▬▬▬▬▬▬▬▬▬▬▬▬▬▬▬▬▬▬▬▬▬▬▬▬▬▬▬▬▬
▬▬▬▬▬▬▬▬▬▬▬▬▬▬▬▬▬▬▬▬▬▬▬▬▬▬▬▬▬▬▬▬▬▬▬▬▬▬▬▬▬▬▬▬▬▬▬▬▬▬▬▬▬
▬▬▬▬▬▬▬▬▬▬▬▬▬▬▬▬▬▬▬▬▬▬▬▬▬▬▬▬▬▬▬▬▬▬▬▬▬▬▬▬▬▬▬▬▬▬▬▬▬▬▬▬▬
▬▬▬▬▬▬▬▬▬▬▬▬▬▬▬▬▬▬▬▬▬▬▬▬▬▬▬▬▬▬▬▬▬▬▬▬▬▬▬▬▬▬▬▬▬▬▬▬▬▬▬▬▬
▬▬▬▬▬▬▬▬▬▬▬▬▬▬▬▬▬▬▬▬▬▬▬▬▬▬▬▬▬▬▬▬▬▬▬▬▬▬▬▬▬▬▬▬▬▬▬▬▬▬▬▬▬
▬▬▬▬▬▬▬▬▬▬▬▬▬▬▬▬▬▬▬▬▬▬▬▬▬▬▬▬▬▬▬▬▬▬▬▬▬▬▬▬▬▬▬▬▬▬▬▬▬▬▬▬▬
▬▬▬▬▬▬▬▬▬▬▬▬▬▬▬▬▬▬▬▬▬▬▬▬▬▬▬▬▬▬▬▬▬▬▬▬▬▬▬▬▬▬▬▬▬▬▬▬▬▬▬▬▬
▬▬▬▬▬▬▬▬▬▬▬▬▬▬▬▬▬▬▬▬▬▬▬▬▬▬▬▬▬▬▬▬▬▬▬▬▬▬▬▬▬▬▬▬▬▬▬▬▬▬▬▬▬
▬▬▬▬▬▬▬▬▬▬▬▬▬▬▬▬▬▬▬▬▬▬▬▬▬▬▬▬▬▬▬▬▬▬▬▬▬▬▬▬▬▬▬▬▬▬▬▬▬▬▬▬▬
▬▬▬▬▬▬▬▬▬▬▬▬▬▬▬▬▬▬▬▬▬▬▬▬▬▬▬▬▬▬▬▬▬▬▬▬▬▬▬▬▬▬▬▬▬▬▬▬▬▬▬▬▬
▬▬▬▬▬▬▬▬▬▬▬▬▬▬▬▬▬▬▬▬▬▬▬▬▬▬▬▬▬▬▬▬▬▬▬▬▬▬▬▬▬▬▬▬▬▬▬▬▬▬▬▬▬
▬▬▬▬▬▬▬▬▬▬▬▬▬▬▬▬▬▬▬▬▬▬▬▬▬▬▬▬▬▬▬▬▬▬▬▬▬▬▬▬▬▬▬▬▬▬▬▬▬▬▬▬▬
▬▬▬▬▬▬▬▬▬▬▬▬▬▬▬▬▬▬▬▬▬▬▬▬▬▬▬▬▬▬▬▬▬▬▬▬▬▬▬▬▬▬▬▬▬▬▬▬▬▬▬▬▬
▬▬▬▬▬▬▬▬▬▬▬▬▬▬▬▬▬▬▬▬▬▬▬▬▬▬▬▬▬▬▬▬▬▬▬▬▬▬▬▬▬▬▬▬▬▬▬▬▬▬▬▬▬
▬▬▬▬▬▬▬▬▬▬▬▬▬▬▬▬▬▬▬▬▬▬▬▬▬▬▬▬▬▬▬▬▬▬▬▬▬▬▬▬▬▬▬▬▬▬▬▬▬▬▬▬▬
▬▬▬▬▬▬▬▬▬▬▬▬▬▬▬▬▬▬▬▬▬▬▬▬▬▬▬▬▬▬▬▬▬▬▬▬▬▬▬▬▬▬▬▬▬▬▬▬▬▬▬▬▬
▬▬▬▬▬▬▬▬▬▬▬▬▬▬▬▬▬▬▬▬▬▬▬▬▬▬▬▬▬▬▬▬▬▬▬▬▬▬▬▬▬▬▬▬▬▬▬▬▬▬▬▬▬
▬▬▬▬▬▬▬▬▬▬▬▬▬▬▬▬▬▬▬▬▬▬▬▬▬▬▬▬▬▬▬▬▬▬▬▬▬▬▬▬▬▬▬▬▬▬▬▬▬▬▬▬▬
▬▬▬▬▬▬▬▬▬▬▬▬▬▬▬▬▬▬▬▬▬▬▬▬▬▬▬▬▬▬▬▬▬▬▬▬▬▬▬▬▬▬▬▬▬▬▬▬▬▬▬▬▬
▬▬▬▬▬▬▬▬▬▬▬▬▬▬▬▬▬▬▬▬▬▬▬▬▬▬▬▬▬▬▬▬▬▬▬▬▬▬▬▬▬▬▬▬▬▬▬▬▬▬▬▬▬
▬▬▬▬▬▬▬▬▬▬▬▬▬▬▬▬▬▬▬▬▬▬▬▬▬▬▬▬▬▬▬▬▬▬▬▬▬▬▬▬▬▬▬▬▬▬▬▬▬▬▬▬▬
▬▬▬▬▬▬▬▬▬▬▬▬▬▬▬▬▬▬▬▬▬▬▬▬▬▬▬▬▬▬▬▬▬▬▬▬▬▬▬▬▬▬▬▬▬▬▬▬▬▬▬▬▬
▬▬▬▬▬▬▬▬▬▬▬▬▬▬▬▬▬▬▬▬▬▬▬▬▬▬▬▬▬▬▬▬▬▬▬▬▬▬▬▬▬▬▬▬▬▬▬▬▬▬▬▬▬
▬▬▬▬▬▬▬▬▬▬▬▬▬▬▬▬▬▬▬▬▬▬▬▬▬▬▬▬▬▬▬▬▬▬▬▬▬▬▬▬▬▬▬▬▬▬▬▬▬▬▬▬▬
▬▬▬▬▬▬▬▬▬▬▬▬▬▬▬▬▬▬▬▬▬▬▬▬▬▬▬▬▬▬▬▬▬▬▬▬▬▬▬▬▬▬▬▬▬▬▬▬▬▬▬▬▬
▬▬▬▬▬▬▬▬▬▬▬▬▬▬▬▬▬▬▬▬▬▬▬▬▬▬▬▬▬▬▬▬▬▬▬▬▬▬▬▬▬▬▬▬▬▬▬▬▬▬▬▬▬

## Slide 9
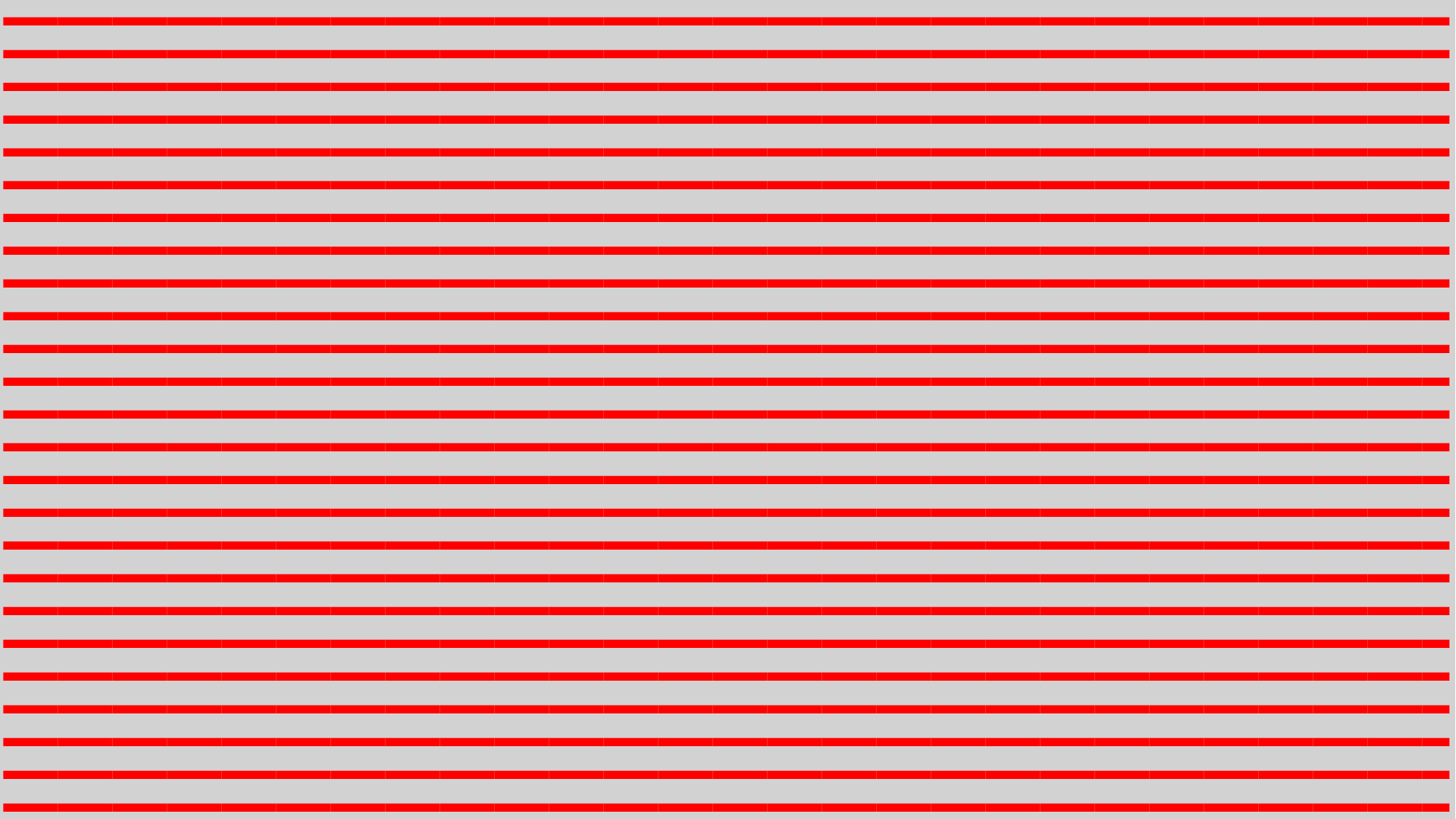

▬▬▬▬▬▬▬▬▬▬▬▬▬▬▬▬▬▬▬▬▬▬▬▬▬▬▬▬▬▬▬▬▬▬▬▬▬▬▬▬▬▬▬▬▬▬▬▬▬▬▬▬▬
▬▬▬▬▬▬▬▬▬▬▬▬▬▬▬▬▬▬▬▬▬▬▬▬▬▬▬▬▬▬▬▬▬▬▬▬▬▬▬▬▬▬▬▬▬▬▬▬▬▬▬▬▬
▬▬▬▬▬▬▬▬▬▬▬▬▬▬▬▬▬▬▬▬▬▬▬▬▬▬▬▬▬▬▬▬▬▬▬▬▬▬▬▬▬▬▬▬▬▬▬▬▬▬▬▬▬
▬▬▬▬▬▬▬▬▬▬▬▬▬▬▬▬▬▬▬▬▬▬▬▬▬▬▬▬▬▬▬▬▬▬▬▬▬▬▬▬▬▬▬▬▬▬▬▬▬▬▬▬▬
▬▬▬▬▬▬▬▬▬▬▬▬▬▬▬▬▬▬▬▬▬▬▬▬▬▬▬▬▬▬▬▬▬▬▬▬▬▬▬▬▬▬▬▬▬▬▬▬▬▬▬▬▬
▬▬▬▬▬▬▬▬▬▬▬▬▬▬▬▬▬▬▬▬▬▬▬▬▬▬▬▬▬▬▬▬▬▬▬▬▬▬▬▬▬▬▬▬▬▬▬▬▬▬▬▬▬
▬▬▬▬▬▬▬▬▬▬▬▬▬▬▬▬▬▬▬▬▬▬▬▬▬▬▬▬▬▬▬▬▬▬▬▬▬▬▬▬▬▬▬▬▬▬▬▬▬▬▬▬▬
▬▬▬▬▬▬▬▬▬▬▬▬▬▬▬▬▬▬▬▬▬▬▬▬▬▬▬▬▬▬▬▬▬▬▬▬▬▬▬▬▬▬▬▬▬▬▬▬▬▬▬▬▬
▬▬▬▬▬▬▬▬▬▬▬▬▬▬▬▬▬▬▬▬▬▬▬▬▬▬▬▬▬▬▬▬▬▬▬▬▬▬▬▬▬▬▬▬▬▬▬▬▬▬▬▬▬
▬▬▬▬▬▬▬▬▬▬▬▬▬▬▬▬▬▬▬▬▬▬▬▬▬▬▬▬▬▬▬▬▬▬▬▬▬▬▬▬▬▬▬▬▬▬▬▬▬▬▬▬▬
▬▬▬▬▬▬▬▬▬▬▬▬▬▬▬▬▬▬▬▬▬▬▬▬▬▬▬▬▬▬▬▬▬▬▬▬▬▬▬▬▬▬▬▬▬▬▬▬▬▬▬▬▬
▬▬▬▬▬▬▬▬▬▬▬▬▬▬▬▬▬▬▬▬▬▬▬▬▬▬▬▬▬▬▬▬▬▬▬▬▬▬▬▬▬▬▬▬▬▬▬▬▬▬▬▬▬
▬▬▬▬▬▬▬▬▬▬▬▬▬▬▬▬▬▬▬▬▬▬▬▬▬▬▬▬▬▬▬▬▬▬▬▬▬▬▬▬▬▬▬▬▬▬▬▬▬▬▬▬▬
▬▬▬▬▬▬▬▬▬▬▬▬▬▬▬▬▬▬▬▬▬▬▬▬▬▬▬▬▬▬▬▬▬▬▬▬▬▬▬▬▬▬▬▬▬▬▬▬▬▬▬▬▬
▬▬▬▬▬▬▬▬▬▬▬▬▬▬▬▬▬▬▬▬▬▬▬▬▬▬▬▬▬▬▬▬▬▬▬▬▬▬▬▬▬▬▬▬▬▬▬▬▬▬▬▬▬
▬▬▬▬▬▬▬▬▬▬▬▬▬▬▬▬▬▬▬▬▬▬▬▬▬▬▬▬▬▬▬▬▬▬▬▬▬▬▬▬▬▬▬▬▬▬▬▬▬▬▬▬▬
▬▬▬▬▬▬▬▬▬▬▬▬▬▬▬▬▬▬▬▬▬▬▬▬▬▬▬▬▬▬▬▬▬▬▬▬▬▬▬▬▬▬▬▬▬▬▬▬▬▬▬▬▬
▬▬▬▬▬▬▬▬▬▬▬▬▬▬▬▬▬▬▬▬▬▬▬▬▬▬▬▬▬▬▬▬▬▬▬▬▬▬▬▬▬▬▬▬▬▬▬▬▬▬▬▬▬
▬▬▬▬▬▬▬▬▬▬▬▬▬▬▬▬▬▬▬▬▬▬▬▬▬▬▬▬▬▬▬▬▬▬▬▬▬▬▬▬▬▬▬▬▬▬▬▬▬▬▬▬▬
▬▬▬▬▬▬▬▬▬▬▬▬▬▬▬▬▬▬▬▬▬▬▬▬▬▬▬▬▬▬▬▬▬▬▬▬▬▬▬▬▬▬▬▬▬▬▬▬▬▬▬▬▬
▬▬▬▬▬▬▬▬▬▬▬▬▬▬▬▬▬▬▬▬▬▬▬▬▬▬▬▬▬▬▬▬▬▬▬▬▬▬▬▬▬▬▬▬▬▬▬▬▬▬▬▬▬
▬▬▬▬▬▬▬▬▬▬▬▬▬▬▬▬▬▬▬▬▬▬▬▬▬▬▬▬▬▬▬▬▬▬▬▬▬▬▬▬▬▬▬▬▬▬▬▬▬▬▬▬▬
▬▬▬▬▬▬▬▬▬▬▬▬▬▬▬▬▬▬▬▬▬▬▬▬▬▬▬▬▬▬▬▬▬▬▬▬▬▬▬▬▬▬▬▬▬▬▬▬▬▬▬▬▬
▬▬▬▬▬▬▬▬▬▬▬▬▬▬▬▬▬▬▬▬▬▬▬▬▬▬▬▬▬▬▬▬▬▬▬▬▬▬▬▬▬▬▬▬▬▬▬▬▬▬▬▬▬
▬▬▬▬▬▬▬▬▬▬▬▬▬▬▬▬▬▬▬▬▬▬▬▬▬▬▬▬▬▬▬▬▬▬▬▬▬▬▬▬▬▬▬▬▬▬▬▬▬▬▬▬▬
▬▬▬▬▬▬▬▬▬▬▬▬▬▬▬▬▬▬▬▬▬▬▬▬▬▬▬▬▬▬▬▬▬▬▬▬▬▬▬▬▬▬▬▬▬▬▬▬▬▬▬▬▬
▬▬▬▬▬▬▬▬▬▬▬▬▬▬▬▬▬▬▬▬▬▬▬▬▬▬▬▬▬▬▬▬▬▬▬▬▬▬▬▬▬▬▬▬▬▬▬▬▬▬▬▬▬
▬▬▬▬▬▬▬▬▬▬▬▬▬▬▬▬▬▬▬▬▬▬▬▬▬▬▬▬▬▬▬▬▬▬▬▬▬▬▬▬▬▬▬▬▬▬▬▬▬▬▬▬▬
▬▬▬▬▬▬▬▬▬▬▬▬▬▬▬▬▬▬▬▬▬▬▬▬▬▬▬▬▬▬▬▬▬▬▬▬▬▬▬▬▬▬▬▬▬▬▬▬▬▬▬▬▬

## Slide 10
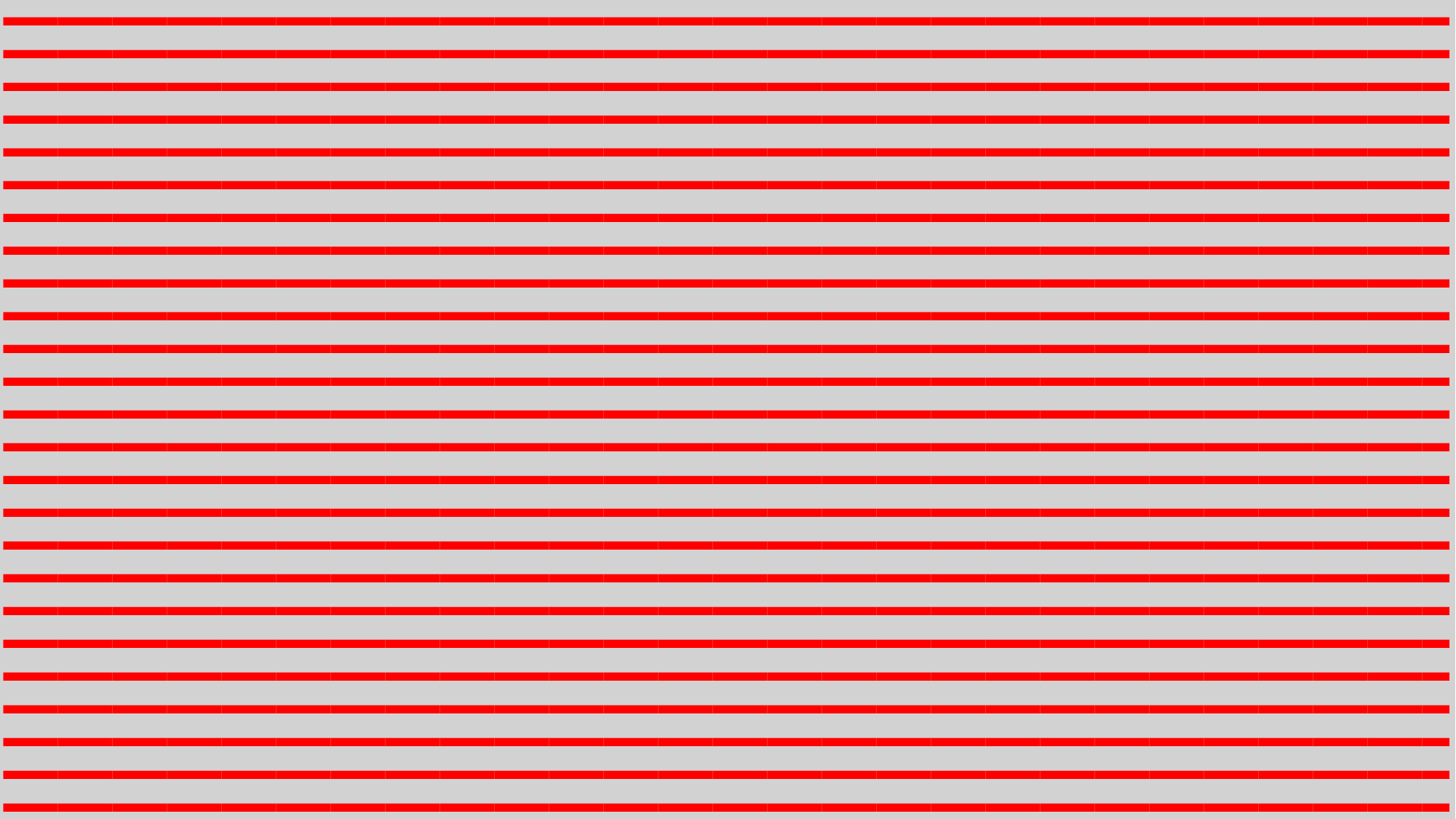

▬▬▬▬▬▬▬▬▬▬▬▬▬▬▬▬▬▬▬▬▬▬▬▬▬▬▬▬▬▬▬▬▬▬▬▬▬▬▬▬▬▬▬▬▬▬▬▬▬▬▬▬▬
▬▬▬▬▬▬▬▬▬▬▬▬▬▬▬▬▬▬▬▬▬▬▬▬▬▬▬▬▬▬▬▬▬▬▬▬▬▬▬▬▬▬▬▬▬▬▬▬▬▬▬▬▬
▬▬▬▬▬▬▬▬▬▬▬▬▬▬▬▬▬▬▬▬▬▬▬▬▬▬▬▬▬▬▬▬▬▬▬▬▬▬▬▬▬▬▬▬▬▬▬▬▬▬▬▬▬
▬▬▬▬▬▬▬▬▬▬▬▬▬▬▬▬▬▬▬▬▬▬▬▬▬▬▬▬▬▬▬▬▬▬▬▬▬▬▬▬▬▬▬▬▬▬▬▬▬▬▬▬▬
▬▬▬▬▬▬▬▬▬▬▬▬▬▬▬▬▬▬▬▬▬▬▬▬▬▬▬▬▬▬▬▬▬▬▬▬▬▬▬▬▬▬▬▬▬▬▬▬▬▬▬▬▬
▬▬▬▬▬▬▬▬▬▬▬▬▬▬▬▬▬▬▬▬▬▬▬▬▬▬▬▬▬▬▬▬▬▬▬▬▬▬▬▬▬▬▬▬▬▬▬▬▬▬▬▬▬
▬▬▬▬▬▬▬▬▬▬▬▬▬▬▬▬▬▬▬▬▬▬▬▬▬▬▬▬▬▬▬▬▬▬▬▬▬▬▬▬▬▬▬▬▬▬▬▬▬▬▬▬▬
▬▬▬▬▬▬▬▬▬▬▬▬▬▬▬▬▬▬▬▬▬▬▬▬▬▬▬▬▬▬▬▬▬▬▬▬▬▬▬▬▬▬▬▬▬▬▬▬▬▬▬▬▬
▬▬▬▬▬▬▬▬▬▬▬▬▬▬▬▬▬▬▬▬▬▬▬▬▬▬▬▬▬▬▬▬▬▬▬▬▬▬▬▬▬▬▬▬▬▬▬▬▬▬▬▬▬
▬▬▬▬▬▬▬▬▬▬▬▬▬▬▬▬▬▬▬▬▬▬▬▬▬▬▬▬▬▬▬▬▬▬▬▬▬▬▬▬▬▬▬▬▬▬▬▬▬▬▬▬▬
▬▬▬▬▬▬▬▬▬▬▬▬▬▬▬▬▬▬▬▬▬▬▬▬▬▬▬▬▬▬▬▬▬▬▬▬▬▬▬▬▬▬▬▬▬▬▬▬▬▬▬▬▬
▬▬▬▬▬▬▬▬▬▬▬▬▬▬▬▬▬▬▬▬▬▬▬▬▬▬▬▬▬▬▬▬▬▬▬▬▬▬▬▬▬▬▬▬▬▬▬▬▬▬▬▬▬
▬▬▬▬▬▬▬▬▬▬▬▬▬▬▬▬▬▬▬▬▬▬▬▬▬▬▬▬▬▬▬▬▬▬▬▬▬▬▬▬▬▬▬▬▬▬▬▬▬▬▬▬▬
▬▬▬▬▬▬▬▬▬▬▬▬▬▬▬▬▬▬▬▬▬▬▬▬▬▬▬▬▬▬▬▬▬▬▬▬▬▬▬▬▬▬▬▬▬▬▬▬▬▬▬▬▬
▬▬▬▬▬▬▬▬▬▬▬▬▬▬▬▬▬▬▬▬▬▬▬▬▬▬▬▬▬▬▬▬▬▬▬▬▬▬▬▬▬▬▬▬▬▬▬▬▬▬▬▬▬
▬▬▬▬▬▬▬▬▬▬▬▬▬▬▬▬▬▬▬▬▬▬▬▬▬▬▬▬▬▬▬▬▬▬▬▬▬▬▬▬▬▬▬▬▬▬▬▬▬▬▬▬▬
▬▬▬▬▬▬▬▬▬▬▬▬▬▬▬▬▬▬▬▬▬▬▬▬▬▬▬▬▬▬▬▬▬▬▬▬▬▬▬▬▬▬▬▬▬▬▬▬▬▬▬▬▬
▬▬▬▬▬▬▬▬▬▬▬▬▬▬▬▬▬▬▬▬▬▬▬▬▬▬▬▬▬▬▬▬▬▬▬▬▬▬▬▬▬▬▬▬▬▬▬▬▬▬▬▬▬
▬▬▬▬▬▬▬▬▬▬▬▬▬▬▬▬▬▬▬▬▬▬▬▬▬▬▬▬▬▬▬▬▬▬▬▬▬▬▬▬▬▬▬▬▬▬▬▬▬▬▬▬▬
▬▬▬▬▬▬▬▬▬▬▬▬▬▬▬▬▬▬▬▬▬▬▬▬▬▬▬▬▬▬▬▬▬▬▬▬▬▬▬▬▬▬▬▬▬▬▬▬▬▬▬▬▬
▬▬▬▬▬▬▬▬▬▬▬▬▬▬▬▬▬▬▬▬▬▬▬▬▬▬▬▬▬▬▬▬▬▬▬▬▬▬▬▬▬▬▬▬▬▬▬▬▬▬▬▬▬
▬▬▬▬▬▬▬▬▬▬▬▬▬▬▬▬▬▬▬▬▬▬▬▬▬▬▬▬▬▬▬▬▬▬▬▬▬▬▬▬▬▬▬▬▬▬▬▬▬▬▬▬▬
▬▬▬▬▬▬▬▬▬▬▬▬▬▬▬▬▬▬▬▬▬▬▬▬▬▬▬▬▬▬▬▬▬▬▬▬▬▬▬▬▬▬▬▬▬▬▬▬▬▬▬▬▬
▬▬▬▬▬▬▬▬▬▬▬▬▬▬▬▬▬▬▬▬▬▬▬▬▬▬▬▬▬▬▬▬▬▬▬▬▬▬▬▬▬▬▬▬▬▬▬▬▬▬▬▬▬
▬▬▬▬▬▬▬▬▬▬▬▬▬▬▬▬▬▬▬▬▬▬▬▬▬▬▬▬▬▬▬▬▬▬▬▬▬▬▬▬▬▬▬▬▬▬▬▬▬▬▬▬▬
▬▬▬▬▬▬▬▬▬▬▬▬▬▬▬▬▬▬▬▬▬▬▬▬▬▬▬▬▬▬▬▬▬▬▬▬▬▬▬▬▬▬▬▬▬▬▬▬▬▬▬▬▬
▬▬▬▬▬▬▬▬▬▬▬▬▬▬▬▬▬▬▬▬▬▬▬▬▬▬▬▬▬▬▬▬▬▬▬▬▬▬▬▬▬▬▬▬▬▬▬▬▬▬▬▬▬
▬▬▬▬▬▬▬▬▬▬▬▬▬▬▬▬▬▬▬▬▬▬▬▬▬▬▬▬▬▬▬▬▬▬▬▬▬▬▬▬▬▬▬▬▬▬▬▬▬▬▬▬▬
▬▬▬▬▬▬▬▬▬▬▬▬▬▬▬▬▬▬▬▬▬▬▬▬▬▬▬▬▬▬▬▬▬▬▬▬▬▬▬▬▬▬▬▬▬▬▬▬▬▬▬▬▬

## Slide 11
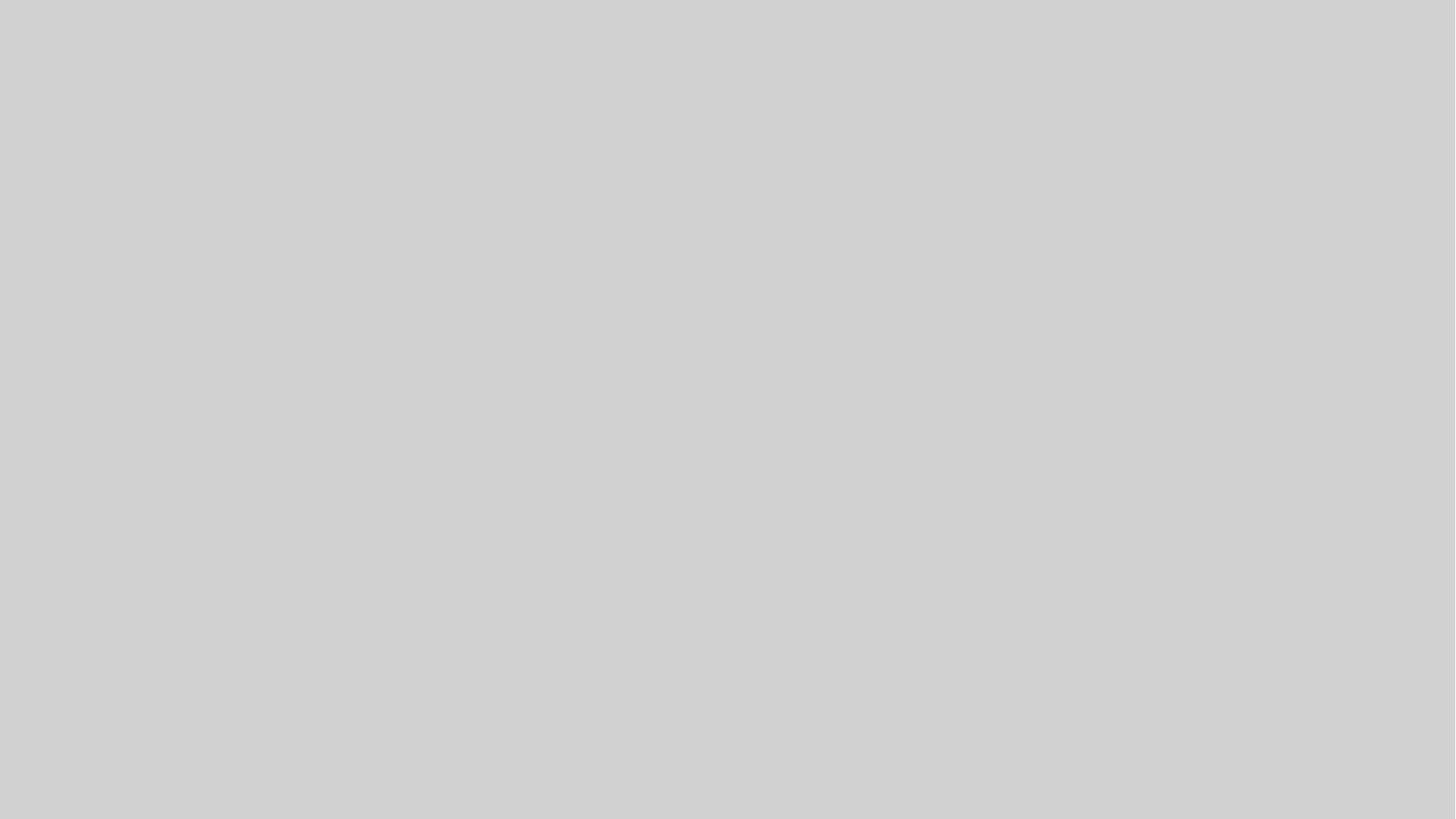

## Slide 12
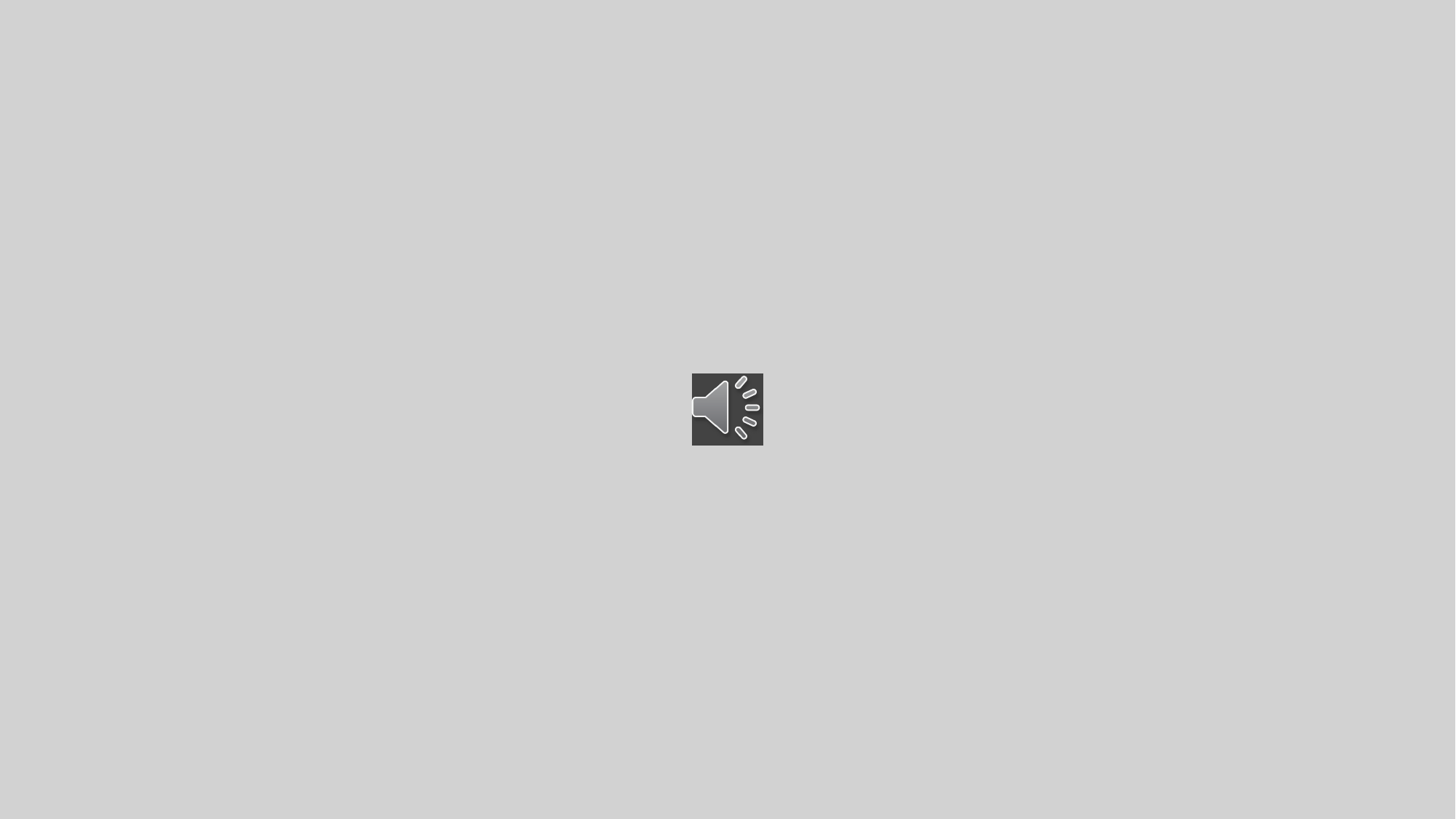

## Slide 13
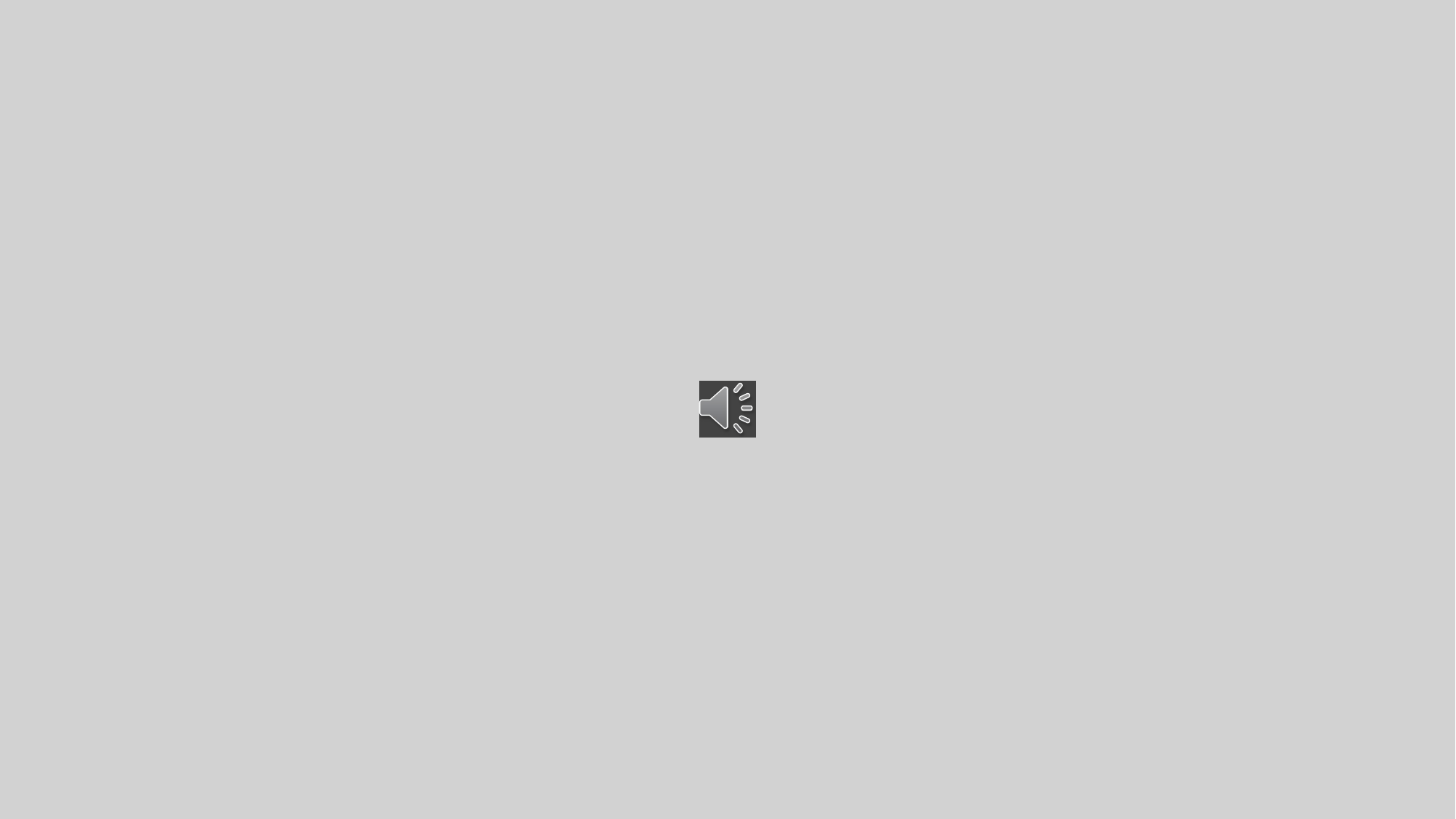

## Slide 14
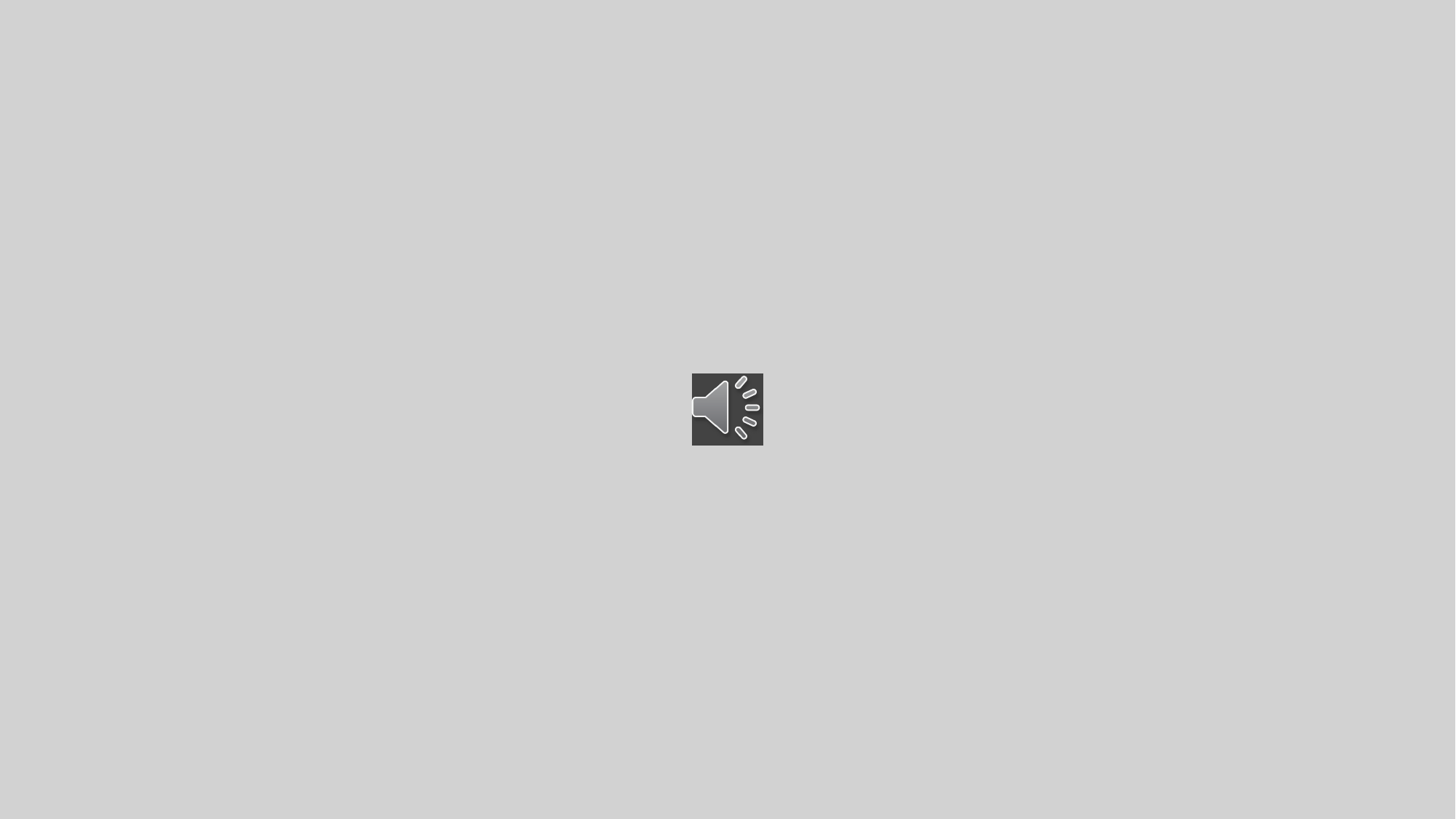

Supplement: Supplementary file 5 — Supplementary Information 5. [file 41598_2023_30303_MOESM5_ESM.pptx]
